# Boceprevir for previously untreated patients with chronic hepatitis C Genotype 1 infection: a US-based cost-effectiveness modeling study

**DOI:** 10.1186/1471-2334-13-190

**Published:** 2013-04-27

**Authors:** Shannon Allen Ferrante, Jagpreet Chhatwal, Clifford A Brass, Antoine C El Khoury, Fred Poordad, Jean-Pierre Bronowicki, Elamin H Elbasha

**Affiliations:** 1 Merck Sharp & Dohme Corp., Whitehouse Station, NJ, USA; 2 Department of Health Policy and Management, University of Pittsburgh Graduate School of Public Health, Pittsburgh, PA, USA; 3 Department of Industrial Engineering, University of Pittsburgh, Pittsburgh, PA, USA; 4 Novartis, East Hanover, NJ, USA; 5 Hepatology and Liver Transplantation, Cedars-Sinai Medical Center, Los Angeles, CA, USA; 6 INSERM 954, Centre Hospitalier Universitaire de Nancy, Université de Lorraine, 54500 Vandoeuvre les Nancy, France

**Keywords:** Cost-effectiveness, Economic evaluation, Hepatitis c virus, Boceprevir

## Abstract

**Background:**

SPRINT-2 demonstrated that boceprevir (BOC), an oral hepatitis C virus (HCV) nonstructural 3 (NS3) protease inhibitor, added to peginterferon alfa-2b (P) and ribavirin (R) significantly increased sustained virologic response rates over PR alone in previously untreated adult patients with chronic HCV genotype 1. We estimated the long-term impact of triple therapy vs. dual therapy on the clinical burden of HCV and performed a cost-effectiveness evaluation.

**Methods:**

A Markov model was used to estimate the incidence of liver complications, discounted costs (2010 US$), quality-adjusted life years (QALYs), and incremental cost-effectiveness ratios (ICERs) of three treatment strategies for treatment-naïve patients with chronic HCV genotype 1. The model simulates the treatment regimens studied in SPRINT-2 in which PR was administered for 4 weeks followed by: 1) placebo plus PR for 44 weeks (PR48); 2) BOC plus PR using response guided therapy (BOC/RGT); and 3) BOC plus PR for 44 weeks (BOC/PR48) and makes projections within and beyond the trial. HCV-related state-transition probabilities, costs, and utilities were obtained from previously published studies. All costs and QALYs were discounted at 3%.

**Results:**

The model projected approximately 38% and 43% relative reductions in the lifetime incidence of liver complications in the BOC/RGT and BOC/PR48 regimens compared with PR48, respectively. Treatment with BOC/RGT is associated with an incremental cost of $10,348 and an increase of 0.62 QALYs compared to treatment with PR48. Treatment with BOC/PR48 is associated with an incremental cost of $35,727 and an increase of 0.65 QALYs compared to treatment with PR48. The ICERs were $16,792/QALY and $55,162/QALY for the boceprevir-based treatment groups compared with PR48, respectively. The ICER for BOC/PR48 compared with BOC/RGT was $807,804.

**Conclusion:**

The boceprevir-based regimens used in the SPRINT-2 trial were projected to substantially reduce the lifetime incidence of liver complications and increase the QALYs in treatment-naive patients with hepatitis C genotype 1. It was also demonstrated that boceprevir-based regimens offer patients the possibility of experiencing great clinical benefit with a shorter duration of therapy. Both boceprevir-based treatment strategies were projected to be cost-effective at a reasonable threshold in the US when compared to treatment with PR48.

## Background

Infection with hepatitis C virus (HCV) is a major global public health problem. According to the World Health Organization statistics, approximately 130–170 million people are currently infected with chronic HCV worldwide [1]. In the United States (U.S.) and Europe, HCV is the leading cause of chronic liver disease and the leading indication for liver transplantation [2-5]. HCV infection represents a substantial clinical and economic burden in the U.S. [6,7]. For example, it is estimated that 3.2 million persons are chronically infected [6] and that HCV infection causes approximately 15,000 deaths annually [8]. The total 2011 healthcare cost associated with HCV in the U.S. was estimated at $6.5 ($4.3–$8.4) billion [9]. Generally, it takes several years – possibly decades – between infection with HCV and development of serious liver disease. Hence, although the incidence of acute HCV infection is declining, the prevalence of cirrhosis and incidence of HCV-related liver disease is expected to increase over the next 10–20 years [10].

There are 6 major HCV genotypes [11]. Approximately 70% of HCV infected people in the U.S. have genotype 1, which is the most difficult-to-treat [12]. Prior to 2011, the standard of care for chronic HCV genotype 1 infection was 48 weeks of antiviral (AV) treatment with a combination of a pegylated interferon alfa and ribavirin [13]. With peginterferon alfa-2a or alfa-2b and ribavirin treatment, less than 50% of treatment-naïve genotype 1 patients achieve a sustained virologic response (SVR) [14,15]. Patients with advanced liver disease and of African-American descent have an even lower likelihood of attaining an SVR with this treatment regimen (20%–30%) [16].

In 2011, HCV protease inhibitors obtained regulatory approval and became available to treat patients infected with HCV genotype 1. The addition of HCV protease inhibitors, boceprevir and telaprevir, to peginterferon alfa and ribavirin have led to markedly higher SVR rates [17-20]. As a result, the American Association for the Study of Liver Diseases (AASLD) guidelines were updated in 2011 to recommend including the protease inhibitors in the treatment regimens of patients infected with HCV genotype 1 [21]. The objective of this study was to assess the clinical impact and cost-effectiveness of the boceprevir-containing regimens that were studied in the Serine Protease Inhibitor Therapy 2 (SPRINT-2) trial in treatment-naïve patients. The secondary objective was to evaluate the cost-effectiveness of boceprevir-based treatment strategies compared to treatment with dual therapy in pre-specified subsets of the population and in sensitivity analyses. The projections are based on a decision analytic model that integrates data from public sources, published literature, and clinical trial databases under a clearly specified set of assumptions.

## Methods

### SPRINT-2 study design

SPRINT-2 (ClinicalTrials.gov number, NCT00705432) was a Phase 3, international, randomized, double-blinded placebo-controlled study comparing the safety and efficacy of therapy with peginterferon alfa-2b and ribavirin (PegIntron and Rebetol, respectively; Merck) with two treatment regimens that added boceprevir (Victrelis, Merck) after a 4-week lead-in treatment period with peginterferon–ribavirin alone [17]. SPRINT-2 was conducted in accordance with the principles of Good Clinical Practice. The study protocol and study design were approved by each of the sites institutional review board and regulatory agencies, and each participant provided written informed consent before undergoing any study-related procedure. A list of the institutional review boards that approved the study protocol and study design is provided in Additional file 1: Table S1. The full study protocol is available at http://www.nejm.org/action/showSupplements?doi=10.1056%2FNEJMoa1010494&viewType=Popup&viewClass=Suppl. Previously untreated patients (N = 1097) ≥18 years of age with genotype 1 chronic HCV and plasma HCV-RNA level ≥10,000 IU/mL were eligible. Because of the known marked difference in SVR rates with peginterferon–ribavirin between black and non-black patients [16], self-identified blacks and non-blacks were enrolled separately into two cohorts. Exclusion criteria included liver disease of other etiology, decompensated cirrhosis, renal insufficiency, HIV or hepatitis B, pregnant/breast feeding women, or active malignancy. Liver biopsies were assigned METAVIR fibrosis and steatosis scores by a single pathologist who was unaware of treatment assignment.

Peginterferon alfa-2b was administered subcutaneously at 1.5 μg/kg once weekly. Ribavirin was administered using weight-based dosing of 600–1400 mg/day (divided daily dose). Boceprevir was administered orally at a dose of 800 mg three times daily (to be taken with food and with an interval of 7 to 9 hours between doses) in four capsules of 200 mg each. Placebo was matched to boceprevir. The study was double-blinded regarding the administration of boceprevir.

All patients received peginterferon–ribavirin during the 4-week lead-in period. Patients randomized to control received peginterferon–ribavirin treatment for 44 weeks after the lead-in period, as well as placebo three times daily beginning at week 5 (PR48). The overall SVR rate in the PR48 arm of SPRINT-2 was 38% (137/366) for both cohorts, 40% (125/311) for the non-black, and 23% (12/52) for the black cohort [17]. Patients randomized to the response-guided therapy (BOC/RGT) regimen received peginterferon–ribavirin plus boceprevir for a total of 24 weeks after the lead-in period; if HCV-RNA levels were undetectable from week 8 through week 24, treatment was considered complete, but if HCV-RNA levels were detectable at any visit from week 8 up to but not including week 24, peginterferon–ribavirin was continued, and placebo was administered at week 28 through week 48. The overall SVR rate in the BOC/RGT arm of SPRINT-2 was 63% for both cohorts (233/368), 67% (211/316) for the non-black, and 42% (22/52) for the black cohort [17]. Patients randomized to the third regimen received peginterferon–ribavirin plus oral boceprevir for 44 weeks after the lead-in period (BOC/PR48). The overall SVR rate in the BOC/PR48 arm of SPRINT-2 was 66% for both cohorts (242/366), 68% (213/311) for the non-black, and 53% (29/55) for the black cohort [17].

In each arm, patients with detectable HCV-RNA at week 24 discontinued treatment as a standard futility rule. Boceprevir was given for 24 weeks in the BOC/RGT arm and 44 weeks in the BOC/PR48 arm. All patients were followed through week 72.

### Model structure

**Figure 1:**
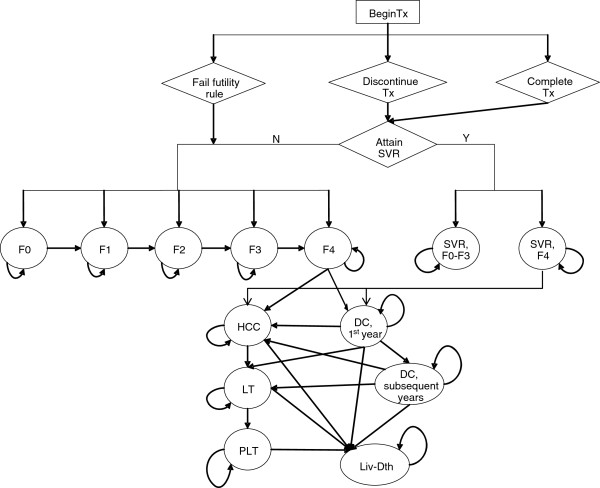
**Schematic Diagram of HCV Therapy and Disease Progression.** Y – yes; N – no; Tx – treatment; ETR – end of treatment response; SVR – sustained virologic response; F0 – no fibrosis; F1 – portal fibrosis without septa; F2 – portal fibrosis with few septa; F3 – numerous septa without cirrhosis; F4 – cirrhosis; DC – decompensated cirrhosis; HCC – hepatocellular carcinoma; LT – liver transplantation; PLT – post-liver transplantation; Lv-death – liver-related death.

We created an Excel-based (Microsoft Corp., Redmond, Washington) Markov model to project health-related outcomes and to estimate the expected costs and quality adjusted life-years (QALYs) associated with the three treatment strategies studied in SPRINT-2. The structure of the model was based on other published health economic models of HCV disease [22-27]. The model consists of two phases: the first phase corresponds to the treatment strategies and follow-up period and the second phase corresponds to post-treatment, which includes the natural history of HCV of cured or uncured patients (Figure 1).

Patients entered the model with chronic HCV and immediately began AV drug therapy. The treatment phase of the model includes a weekly cycle length in which patients can stop therapy early for a variety of reason (i.e. discontinued due to standard futility rule, adverse events, or other non-medical reasons). Patients with undetectable HCV-RNA at the end of treatment were followed for an additional 24 weeks. Patients with undetectable HCV-RNA after 24 weeks of follow-up achieved an SVR. Relapse was defined as the occurrence of undetectable HCV-RNA at the end of treatment, but detectable HCV-RNA after the 24 week follow-up period. In the model, patients who experienced relapse returned to the chronic HCV health states. Patients who failed to pass a futility rule or who had detectable HCV-RNA at the end of treatment were considered treatment failures and also returned to the chronic HCV health states.

The second phase of the model uses cycles with a length of one year and describes patient outcome post-treatment. After the trial follow-up period (72 weeks), patients either attained an SVR or returned to the natural health states of HCV. The severity of chronic HCV infection was defined by the degree of fibrosis using the METAVIR scoring system: no fibrosis (F0), portal fibrosis without septa (F1), portal fibrosis with few septa (F2), numerous septa without fibrosis (F3), and cirrhosis (F4). Patients with mild or moderate chronic HCV at baseline, described by a METAVIR fibrosis score of F0*-*F3, who attained an SVR were considered permanently cured; cirrhotic patients who attained an SVR were considered partially cured. Patients who were permanently cured were considered to be neither at risk for developing further HCV-related liver complications nor for reinfection. Patients with cirrhosis at baseline continued to be at risk for developing advanced stages of liver complications associated with cirrhosis even if they achieved SVR; however, for cirrhotic patients who attained an SVR the probability of developing advanced stages of liver complications was less than that of an untreated cirrhotic patient [28].

Patients who failed to achieve an SVR returned to the natural HCV health states and were at risk for developing serious liver disease and could receive a liver transplant at the same rate as patients who did not receive treatment. The model simulates the natural history of chronic HCV and advanced liver-related diseases and its treatment, consistent with the current understanding of the biology of chronic HCV-related liver disease and its treatment (e.g., liver transplant). The progressive disease model assumed that during one cycle, a person with a given fibrosis score could progress to the next fibrosis level of severity or could remain in that current health state. In the absence of successful treatment, regression to less severe health states, or spontaneous clearance of the virus, was not permitted. Patients with compensated cirrhosis were at risk for developing the sequelae of cirrhosis - decompensated cirrhosis (DC) and hepatocellular carcinoma (HCC).

Patients who developed DC and/or HCC could receive a liver transplant. Because of differential mortality, costs and quality of life between the first year and subsequent years of these health states, they were divided into two states: DC, first year and DC, subsequent years; HCC, first year and HCC, subsequent years; and the Liver Transplant and Post-Liver Transplant states. The Liver Transplant health state lasted a total of 1 year. If a patient was alive at the end of 1 year, then the patient transitioned to the Post-Liver Transplant state. Patients who received a liver transplant were assumed to be at no risk of reactivation and progression to liver disease.

The mortality risk of the general population was applied to all states in the model. In addition, an excess mortality rate was applied to patients with DC, HCC, or who received a liver transplant.

### Model inputs

The model required information describing the patient characteristics for the treatment population as well as model inputs describing treatment characteristics, clinical inputs, costs and utility values associated with AV therapy and the HCV health states (Tables 1, 2 and 3). The patient characteristics of the cohorts and the treatment characteristics were obtained from SPRINT-2. Baseline values and plausible ranges to be used in deterministic and probabilistic sensitivity analyses for model inputs describing the clinical characteristics of HCV and the utility values applied to each of the health states were obtained from published studies. All clinical inputs used in the model are summarized in Table 3.

**Table 1:** **Selected baseline characteristic of patients enrolled in SPRINT-2, all treatment arms combined **[17]

**Characteristics**	**Combined cohorts (N = 1097)**
Race Cohort, no. (%)	
Black	159 (14.5)
Non-Black	938 (85.5)
Age, mean (standard deviation), years	49.1 (9.4)
Male sex, no. (%)	656 (60)
METAVIR Score, without missing data, no (%)	N = 1060
F0 – no fibrosis	47 (4.4)
F1 – portal fibrosis without septa,	730 (68.9)
F2 – portal fibrosis with few septa	183 (17.3)
F3 – numerous septa without cirrhosis	47 (4.4)
F4 – cirrhosis	53 (5)

**Table 2:** **Treatment characteristics from SPRINT-2 **[17]

**A. Efficacy and Discontinuation Rates**						
	**Non-Black Cohort**			**Black Cohort**		
	**PR48**	**BOC/RGT**	**BOC/PR48**	**PR48**	**BOC/RGT**	**BOC/PR48**
	**(N = 311)**	**(N = 316)**	**(N = 311)**	**(N = 52)**	**(N = 52)**	**(N = 55)**
Sustained virologic response,% (95% confidence interval) distribution	40.2 (34.7–45.9)	66.8 (61.3–71.9)	68.5 (63.0–73.6)	23.1 (12.5–45.9)	42.3 (28.7–56.8)	52.7 (38.8–66.3)
	Beta (123.00, 183.02)	Beta (212.7, 105.85)	Beta (216.12, 99.43)	Beta (123.00, 183.02)	Beta (212.7, 105.85)	Beta (216.12, 99.43)
Probability of discontinuation before Week 24 for reasons other than futility, n/m (%)	46/311 (14.8)	49/316 (15.5)	46/311 (14.8)	10/52 (19.2)	12/52 (23.5)	8/55 (14.5)
Probability of discontinuation after Week 24 for reasons other than futility, n/m (%)	25/173 (14.5)	20/225 (8.9)	42/232 (18.1)	6/17 (33.3)	3/27 (11.1)	8/33 (22.9)
Probability of failing futility rule at Week 24, n/m (%)	92/265 (34.7)	42/267 (15.7)	33/265 (12.5)	25/42 (59.5)	13/40 (32.5)	14/47 (29.8)
Probability of being assigned and completing 28 weeks of treatment, n (%)	NA	147 (46.5)	NA	NA	15 (28.8)	NA
**B. Side Effects**	**Combined Cohorts**					
	**PR48**		**BOC/RGT**		**BOC/PR48**	
	**(N = 363)**		**(N = 368)**		**(N = 366)**	
Anemia, n (%)	107 (29.5)		182 (49.5)		180 (49.2)	
Erythropoietin use, n (%)	87 (24.0)		159 (43.2)		159 (43.4)	
Mean duration of anemia, Days	128.3		107.9		145.0	
Mean duration of erythropoietin use, days	121.4		93.5		156.4	

PR48 – peginterferon-ribavirin regimen for 48 weeks; BOC/RGT – peginterferon-ribavirin and boceprevir for 24 weeks, and those with a detectable hepatitis C virus (HCV) RNA level between weeks 8 and 24 received peginterferon–ribavirin from week 28 to week 48; BOC/PR48 –peginterferon–ribavirin for 48 weeks and boceprevir for 44 weeks.

**Table 3:** Clinical inputs

**A. Annual transition probabilities (source)**			**Baseline (Range)**	**PSA Distribution (Parameter1, parameter2)**
Fibrosis progression				
F0 to F1 [29]			0.117 (0.104–0.130)	Beta (274.98, 2075.30)
F1 to F2 [29]			0.085 (0.075–0.096)	Beta (210.06, 2261.18)
F2 to F3 [29]			0.120 (0.109–0.133)	Beta (288.05, 2112.38)
F3 to F4/Compensated Cirrhosis [29]			0.116 (0.104–0.129)	Beta (270.61, 2062.22)
F4 to DC [30-34]			0.029 (0.020–0.083)	Beta (16.67, 558.01)
F4 to HCC [30-38]			0.028 (0.010–0.044)	Beta (22.97, 791.67)
DC to HCC [39]			0.068 (0.030–0.083)	Beta (10.88, 149.15)
SVR, F4 to DC [28]			0.008	Beta (6348.80, 787251.20)
SVR, F4 to HCC [28]			0.005	Beta (2487.50, 495012.50)
Probability of Receiving a Liver Transplant				
DC [40-42]			0.023 (0.010–0.062)	Beta (1.31, 55.44)
HCC [43]			0.040 (0.000–0.140)	Beta (3.88, 93.09)
Mortality Rates				
All-Cause mortality [44]			age/gender specific	NA
Liver-related mortality associated with DC, first year [39]			0.142 (0.065–0.190)	Beta (68.42, 307.52)
Liver-related mortality associated with DC, subsequent years [39]			0.112 (0.065–0.190)	Beta (28.13, 223.02)
Liver-related mortality associated with HCC [30]			0.427 (0.330–0.860)	Beta (263.82, 354.02)
Mortality associated with liver transplant [45]			0.116 (0.060–0.420)	Beta (30.04, 228.91)
Mortality associated with post-liver transplant [45]			0.044 (0.024–0.110)	Beta (4.67, 101.55)
**B. Economic and Health Related Utilities Inputs**				
	**Weekly Costs ($)**		**Utilities**	
	**Baseline (Range)**	**Distribution**	**Baseline (Range)**	**Distribution**
Pegylated Interferon [46]	588	NA	NA	NA
Ribavirin [46]	309	NA	NA	NA
Boceprevir [46]	1,100	NA	NA	NA
Erythropoietin [46]	875	NA	NA	NA
Monitoring Costs [26]	64	NA	NA	NA
AV Therapy, No Anemia [24]	NA	NA	0.90 (0.84, 0.96)	NA
AV Therapy, Anemia [47]	NA	NA	0.83 (0.75, 0.97)	NA
US population norms [48]	NA	NA	Age/gender specific	Beta
	Annual Costs ($)		Utilities	
SVR, F0–F4	0 (0, 509)	NA	1.00 (0.92, 1.00)	Beta (6368.04, 15.96)
F0, F1 [49-51]	678 (509, 848)	Gamma (61.47, 11.03)	0.93 (0.84, 1.00)	Beta (47.47, 3.57)
F2 [49-51]	687 (515, 859)	Gamma (61.47, 11.17)	0.93 (0.84, 1.00)	Beta (47.47, 3.57)
F3 [49-51]	1,394 (1045, 1742)	Gamma (61.47, 22.67)	0.93 (0.84, 1.00)	Beta (47.47, 3.57)
F4 [49,51]	1,626 (1220, 2033)	Gamma (61.47, 26.46)	0.90 (0.81, 1.00)	Beta (31.12, 3.46)
DC [49,51]	18,064 (13548, 22580)	Gamma (61.47, 293.89)	0.80 (0.57, 1.00)	Beta (12.29, 3.07)
HCC [49,51]	33,218 (24914, 41523)	Gamma (61.47, 540.44)	0.79 (0.54, 1.00)	Beta (11.42, 3.03)
Liver Transplantation [49,51]	95,971 (71979, 119964)	Gamma (61.47, 1561.38)	0.84 (0.77, 0.93)	Beta (53.54, 10.20)
Post-Liver Transplantation [49,51]	25,208 (18906, 31510)	Gamma (61.47, 410.11)	0.84 (0.77, 0.93)	Beta (53.54, 10.20)

SVR – sustained virologic response; F0 – no fibrosis; F1 – portal fibrosis without septa; F2 – portal fibrosis with few septa; F3 – numerous septa without cirrhosis; F4 – cirrhosis; DC – decompensated cirrhosis; HCC – hepatocellular carcinoma; AV therapy – antiviral therapy.

### Patient characteristics

As patient characteristics impact the efficacy of the treatment regimens and the annual mortality rate, the analyses were conducted on a cohort of persons with chronic HCV genotype 1 who were representative of participants in SPRINT-2. In the model, a series of 20 cohorts progressed through each treatment regimen. The cohorts represent all possible combinations of gender, race cohort, and baseline METAVIR fibrosis score (2 × 2 × 5 = 20). The average age of the overall SPRINT-2 study cohort was applied to all patients in the analysis. The reported distributions of gender, baseline fibrosis level, and race cohort from SPRINT-2 were assumed for the treatment population (17, Table 1).

### Treatment characteristics

Treatment characteristics were obtained from reported data in SPRINT-2 (Table 2). We assumed that patients experienced the same treatment efficacy, discontinuation rates, treatment-related anemia, and utilized erythropoietin (EPO) to treat anemia as observed in SPRINT-2.

### Clinical inputs

Clinical inputs described the rate of HCV progression, the probability of receiving a liver transplant, and all-cause and liver-related mortality rates and were used in the model to determine the amount of time patients spend in each HCV health state (Table 3).

### Progression of HCV infection

The literature search resulted in a wide range of values for progression rates [52,53]. We used the progression rates from Thein et al. [29], a recent meta-analysis of published progression rates from 111 studies of individuals with chronic HCV infection. They provided stage-specific progression rates by fibrosis level, as described using the METAVIR scoring system. The meta-analysis demonstrated that the progression rates are not linear and are generally higher in the initial stage F0 to F1 than the transitions between the stages with more fibrosis (i.e. F1 to F2). The estimates were also adjusted for biases attributable to study design and selection factors associated with study population and clinical characteristics.

The annual probability of developing advanced stages of liver complications associated with cirrhosis was derived from published studies. The baseline likelihood of developing DC from compensated cirrhosis was estimated using a weighted average of the annual incidence rates reported in five natural history studies of 1,276 cirrhotic patients [30-34]. Similarly, the annual transition rate from compensated cirrhosis to HCC was estimated using a weighted average of the annual incidence rates reported in nine natural history studies of 1,905 cirrhotic patients [30-38]. The baseline likelihood of developing HCC from DC was estimated from a study by Planas et al. [39] that followed 200 patients with DC. The estimates for the transition rates to DC and HCC in cirrhotic patients who achieved SVR were obtained from a study by Cardoso et al. [28].

### Probability of receiving a liver transplant

Previously published U.S. based cost-effectiveness models estimated the probability of receiving a liver transplant from DC using Bennett et al. [54], which estimated the prevalence of DC using mortality rates from a 1987 study by Gines et al. [55] and 1994 data from United Network of Organ Sharing (UNOS) and the Division of Organ Transplantation [40]. In our model, this estimate was updated to take into account the increase in survival rates, prevalence of patients with liver decompensation, and changes in liver-transplant practice that have occurred since 1994. According to the analysis of the Scientific Registry of Liver Transplant Recipients (SRTR) data, from 1999 to 2007, the number of recipients with HCV increased to a peak of 2,481 in 2006 and remained relatively stable at around 2,400 transplants annually thereafter [41]. In addition, HCV-related DC became more common after 1995 with the prevalence in 2010 estimated as 103,117 [42]. We estimated the annual probability of receiving a liver transplant from DC by dividing the most recent data for the number of HCV-related liver-transplants with the prevalence of HCV-related DC, i.e. 2400/103117 = 2.3%. Although the number of liver transplants has increased, our estimate is lower than that assumed in previous studies (3.1%) primarily because of a substantial increase in the prevalence of DC. The annual probability of patients with HCC receiving a liver transplant was estimated as 4.0% using a study by Lang et al. [43].

### Mortality

We used gender and age-specific all-cause mortality rates from the 2006 U.S. life tables to describe the risk of mortality associated with all states in the model [44]. In addition, an excess mortality rate associated with decompensation of the liver was estimated from Planas et al. [39] and probability of death from HCC was obtained from Fattovich et al. [30]. Liver-transplant related mortality was estimated from the most recent data available on liver transplants using the study by Wolfe et al. [45].

### Cost inputs

The model was developed from the payer perspective. We included the cost of AV therapy and management of HCV disease in patients who did not achieve SVR. All costs were expressed in terms of 2010 US dollars. AV therapy costs include drug costs, the cost of managing treatment-related anemia, and monitoring costs for patients on therapy. AV drug costs were calculated using the weekly costs of peginterferon and the generic version of ribavirin, assuming a daily dose of 1000 mg [46]. The weekly cost of boceprevir was assumed to be $1100. The drug costs accounted for the discontinuation of treatment due to the standard futility rule, adverse events, or other non-medical reasons. As ribavirin is administered using weight-based dosing, the average weight of patients was used to determine the cost of ribavirin.

The average costs applied to each natural HCV health state were derived from published studies. The costs of treating chronic HCV and compensated cirrhosis were based on a retrospective, matched cohort claims database study [49]. The reported costs were modified by subtracting the AV therapy costs reported by patients with a METAVIR score of F0–F4 and by adjusting the inpatient hospitalization costs using the national hospital cost-to-charge ratio. McAdam-Marx et al. [49] only reported an aggregated cost for the F0–F3 health states. The disaggregated costs of ''HCV without liver disease" were estimated by taking into account the relative contribution of mild (F0, F1), moderate (F2), and severe (F3) to the total HCV-related cost reported in Davis et al. [50]. Future costs were discounted at 3% per year.

### Quality of life inputs

Utility weights for each of the health states and liver disease conditions were applied to the utilities of the general population. The utility weights were estimated from a previously published study of patients with chronic HCV [51] and adjusted to the U.S. population norm by using age- and gender-specific utility weights of the U.S. general population [48]. There are limited data on the impact of treatment on health-related quality of life. Side effects associated with pegylated interferon and ribavirin are well-documented and include IFN-induced bone marrow depression, flu‒like symptoms, neuropsychiatric disorders, autoimmune syndromes, and anemia [56]. In addition to these side effects, the boceprevir-based regimens were also associated with a higher probability of anemia and dysguesia [17]. The magnitude of the decrement in quality of life associated with the side effects of boceprevir has not been empirically quantified. In our analysis, we assumed that patients who do not experience side effects experienced the same decrement in quality of life regardless of their treatment strategy (PR48 vs. BOC/RGT and BOC/PR48). Similarly, we assumed that patients with incident anemia experienced the same decrement in quality of life regardless of their treatment strategy. We used previously published estimates to quantify the impact of AV therapy on the quality of life for patients with chronic HCV without incident anemia [24]. The impact of incident anemia on the quality of life for patients who are receiving AV treatment for HCV was estimated from a study of the impact of anemia on the quality of life of patients with cancer [47]. Differences in the average quality of life amongst the regimens for patients on AV therapy are due to differences in the proportion of patients experienced anemia.

For patients who are cured by treatment, we assumed that an SVR following treatment eliminates all decrements in health-related quality of life associated with living in the chronic HCV infection state. Future QALYs were discounted at 3% per year.

### Model outcomes

The model was run for each of the specified 20 patient profiles. An overall weighted average of the results was generated based on the distribution of the patient characteristics assumed for a given analysis. The model projected the lifetime incidence of serious liver complications, total costs, and QALYs associated with each treatment strategy as well as no treatment. In addition, the incremental cost-effectiveness ratios (ICERs) of boceprevir-based regimens compared with treatment with peginterferon and ribavirin alone as well as for PR48 compared with no treatment were estimated.

### Sensitivity and subset analyses

Extensive sensitivity analyses on inputs were performed to estimate their impact on the total costs and QALYs of the boceprevir-based regimens and the dual therapy regimen. Specifically, we examined the effect of varying the values of inputs related to SVR rates, progression rates, cost, and quality of life weights in both one-way and multivariate sensitivity analyses. In the sensitivity analyses, the lower and upper bounds of the transition rates and QALYs were obtained from published studies; the costs were varied by increasing and decreasing the base case values by 25%; and the lower and upper bounds of the SVR rates were obtained from the bounds of their respective 95% confidence intervals on the reported values. In the multivariate sensitivity analyses, we applied the lower and upper bounds of all similar variables (i.e. the lower bounds of all progression rates). In addition, a probabilistic sensitivity analysis (PSA) was conducted to examine the impact of varying these covariates simultaneously. The baseline values, range of values examined in sensitivity analyses, distribution of parameters assumed for the PSA, and references for clinical, economic, and utility parameters are included in Table 3. The baseline values, range of values examined in sensitivity analyses, and distribution of parameters assumed for the PSA for the treatment efficacy rates are included in Table 2. The results of the PSA were based on 10,000 Monte Carlo simulation runs.

Patients in SPRINT-2 were randomized by race cohort to each of the three treatment groups and the data was analyzed separately in the primary efficacy analysis. Hence, subgroup analyses were generated for each race cohort (non-black vs. black). Because the recommended treatment regimen for boceprevir use in the U.S. is not exactly as was studied in the trials, we conducted a supplemental analysis depicting the Food and Drug Administration (FDA)-approved label [57] recommendations by modifying the treatment strategy phase in the model and reanalyzing the data from SPRINT-2. The label includes different treatment strategies for patients without cirrhosis and patients with cirrhosis. The recommended treatment strategy for patients without cirrhosis is similar to the BOC/RGT arm in SPRINT-2 and the recommended treatment strategy for patients with cirrhosis is similar to the BOC/PR48 arm in SPRINT-2. Model modifications include estimating the treatment characteristics for patients based on their baseline cirrhosis status (METAVIR score of F0–F3 vs. F4), and accounting for an additional futility rule at week 12.

## Results

### No treatment compared with dual therapy

Over the lifetime of this cohort, our model predicted that treatment with PR48 will result in relative decreases in the cumulative incidence by 37% in DC, 38% in HCC, 38% in liver transplants, and 38% in liver-related deaths compared to no treatment. The total discounted lifetime costs and QALYs associated with no treatment are $37,230 and 13.67, respectively. The total discounted lifetime costs and QALYs associated with PR48 treatment are $58,761 and 14.55, respectively. The corresponding ICER comparing PR48 treatment with no treatment is $24,435/QALY.

### Base case analysis

**Figure 2:**
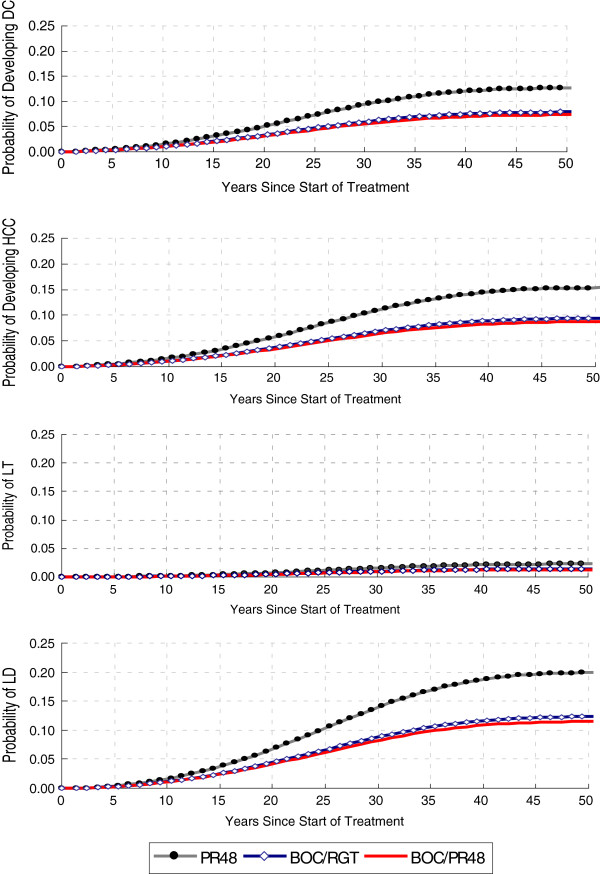
**Cumulative risk of developing HCV liver-related complications, by SPRINT-2 Treatment Strategy, over time.** DC – decompensated cirrhosis; HCC – hepatocellular carcinoma; LT – liver transplantation; LD – liver-related death; PR48 – peginterferon-ribavirin regimen for 48 weeks; BOC/RGT – peginterferon-ribavirin and boceprevir for 24 weeks, and those with a detectable hepatitis C virus (HCV) RNA level between weeks 8 and 24 received peginterferon–ribavirin from week 28 to week 48; BOC/PR48 –peginterferon–ribavirin for 48 weeks and boceprevir for 44 weeks.

The model projected the lifetime cumulative risk of developing HCV-related liver complications associated with each of the treatment strategies studied in SPRINT-2 over time (Figure 2). Over the lifetime of this cohort, our model predicted that treatment with BOC/RGT will result in relative decreases in the cumulative incidence by 38% in DC, 39% in HCC, 38% in liver transplants, and 38% in liver-related deaths compared to treatment with PR48. This implies that treating 21 patients with BOC/RGT instead of PR48 will avoid 1 case of DC; treating 17 patients will avoid 1 case of HCC; treating 116 patients will avoid 1 liver transplant; and treating 13 patients will avoid 1 liver-related death. Similarly, our model predicted treatment with BOC/PR48 will result in relative decreases in the cumulative incidence by 42% in DC, 43% in HCC, 42% in liver transplants, and 42% in liver-related deaths compared to treatment with PR48. This implies that treating 19 patients with BOC/PR48 instead of PR48 will avoid 1 case of DC; treating 15 patients will avoid 1 case of HCC; treating 104 patients will avoid 1 liver transplant; and treating 12 patients will avoid 1 liver-related death. In addition, treatment with BOC/RGT and treatment with BOC/PR48 are associated with overall increases in life expectancy of 0.97 and 1.07 years, respectively, when compared with PR48 treatment.

The total discounted lifetime costs and QALYs associated with each treatment strategy are summarized in Table 4. The ICERs of both boceprevir-based regimens were calculated in comparison with the PR48 treatment arm. Although the AV therapy costs of BOC/RGT and BOC/PR48 are considerably higher ($47,582 and $69,928) than the AV therapy costs of PR48 ($29,573), the projected costs of managing HCV and HCV-related liver disease in patients who received boceprevir-based treatment were 37%–42% lower than that in patients who received PR48. Compared to treatment with PR48, the ICER for treatment with BOC/RGT was $16,792/QALY and the ICER for treatment with BOC/PR48 was $55,162/QALY. The ICER for treatment with BOC/PR48 compared with BOC/RGT was $807,804/QALY.

**Table 4:** Base-case cost-effectiveness results (per patient): discounted lifetime costs, QALYs and incremental cost-effectiveness ratios of BOC/RGT vs. PR48 and BOC/PR48 vs. PR48

	**PR48**	**BOC/RGT**	**BOC/PR48**
Costs (2010 US$):			
AV Therapy Drug Costs	29,573	47,582	69,928
EPO for treatment-related anemia	3,637	5,050	8,493
Monitoring Costs	2,110	1,796	2,380
SVR	0	0	0
F0-F3	7,538	4,786	4,461
Compensated Cirrhosis, F4	3,749	2,266	2,100
Decompensated Cirrhosis	4,223	2,677	2,505
Hepatocellular Carcinoma	5,043	3,128	2,915
Liver Transplantation	1,067	669	624
Post-Liver Transplant	1,822	1,155	1,081
Total Costs	**58,761**	**69,110**	**94,488**
Total QALYs	**14.55**	**15.17**	**15.20**
ICER		16,792/QALY	55,162/QALY

AV therapy – antiviral therapy; SVR – sustained virologic response; F0 – no fibrosis; F1 – portal fibrosis without septa; F2 – portal fibrosis with few septa; F3 – numerous septa without cirrhosis; F4 – cirrhosis; QALY – quality-adjusted life years; ICER – incremental cost-effectiveness ratios; PR48 – peginterferon-ribavirin regimen for 48 weeks; BOC/RGT – peginterferon–ribavirin for 4 weeks followed by peginterferon-ribavirin and boceprevir for 24 weeks, and those with a detectable hepatitis C virus (HCV) RNA level between weeks 8 and 24 received peginterferon–ribavirin from week 28 to week 48; BOC/PR48 –peginterferon–ribavirin for 48 weeks and boceprevir for 44 weeks.

### Sensitivity analyses

The ICERs compared with PR48 from the one-way sensitivity analyses of chronic disease progression rates, rate of developing advanced liver disease, all health state costs, and most utility values were within $6 K/QALY and $11 K/QALY of the BOC/RGT (range: $1,747 to $42,983/QALY) and BOC/PR48 (range: $21,016 to $88,789/QALY) base-case ICERs, respectively (See Additional file 2: Table S2 online). The ICERs that fell out of these ranges were obtained when the lower bound of the quality of life of the SVR state for patients who had a baseline METAVIR score of F1 was assumed (BOC/RGT: $25,685 and BOC/PR48: $87,264) and when assumptions concerning treatment efficacies were varied. When the efficacy of PR48 was assumed to be 45.4%, the upper limit of the 95% confidence bound, and the efficacies for BOC/RGT and BOC/PR48 remained the base case values, the ICERs of BOC/RGT and BOC/PR48 increased to $29,369 and $81,237, respectively. Conversely, when the efficacies of the boceprevir-based regimens were assumed to be the upper limits of the confidence bounds, and the efficacy of PR48 was assumed to be the base case value, both BOC/RGT and BOC/PR48 became cost-saving compared to dual therapy.

Compared to treatment with PR48, the ICERs from the multivariate sensitivity analyses ranged from $2,338 to $33,511 for BOC/RGT and from $21,016 to $117,395 for BOC/PR48 (Table 4). The ICERs are most sensitive to assumptions concerning the quality of life of the HCV health states (range: $10,906–$31,124/QALY for BOC/RGT vs. PR48; range: $34,927–$108,965/QALY for BOC/PR48 vs. PR48) and least sensitive to assumptions concerning the quality of life of patients on treatment for those who receive BOC/RGT (range: $16,724–$16,819/QALY) and quality of life of the general population for patients who receive BOC/PR48 (range: $54,133–$56,228/QALY).

**Figure 3:**
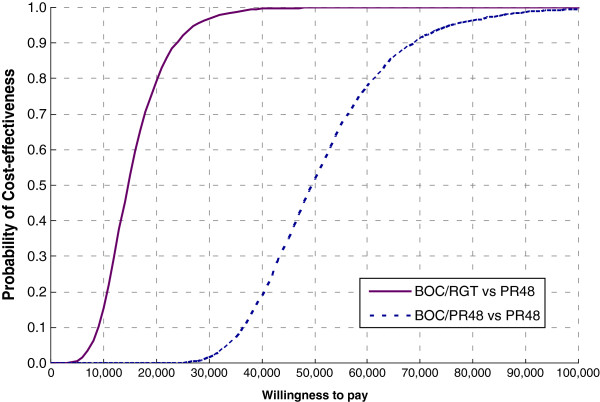
**Cost-effectiveness acceptability curve of SPRINT-2 treatment strategies.** BOC/RGT vs. PR48 and BOC/PR48 vs. PR48. PR48 – peginterferon-ribavirin regimen for 48 weeks; BOC/RGT – peginterferon-ribavirin and boceprevir for 24 weeks, and those with a detectable hepatitis C virus (HCV) RNA level between weeks 8 and 24 received peginterferon–ribavirin from week 28 to week 48; BOC/PR48 –peginterferon–ribavirin for 48 weeks and boceprevir for 44 weeks.

The results of the corresponding probabilistic sensitivity analysis are described in the cost-effectiveness acceptability curves (Figure 3). Compared to treatment with PR48, and using an incremental cost-effectiveness ratio of $50,000 per QALY as a threshold, treatment with BOC/RGT was cost-effective in 99.9% of the simulations and treatment with BOC/PR48 was cost-effective in 51.9% of the simulations. Using an incremental cost-effectiveness ratio of $100,000 per QALY as a threshold, treatment with BOC/RGT was cost-effective in 100% of the simulations and treatment with BOC/PR48 was cost-effective in 99.5% of the simulations.

### Subset analyses

**Figure 4:**
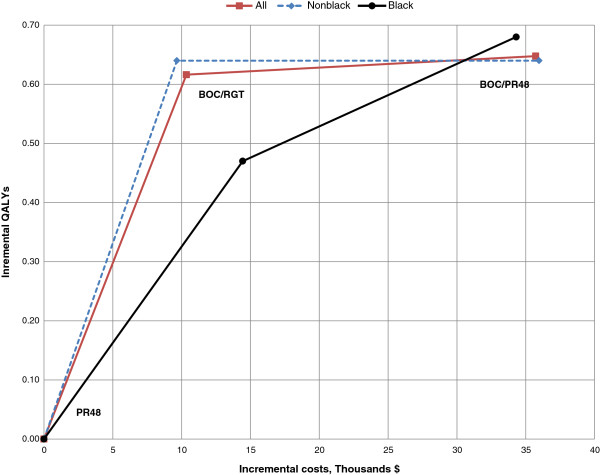
**Cost-effectiveness frontier of SPRINT-2 treatment strategies.** BOC/RGT vs. PR48 and BOC/PR48 vs. PR48 for all patients and by race cohort. PR48 – peginterferon-ribavirin regimen for 48 weeks; BOC/RGT – peginterferon-ribavirin and boceprevir for 24 weeks, and those with a detectable hepatitis C virus (HCV) RNA level between weeks 8 and 24 received peginterferon–ribavirin from week 28 to week 48; BOC/PR48 –peginterferon–ribavirin for 48 weeks and boceprevir for 44 weeks.

In the non-black cohort, treatment with both boceprevir-based regimens were projected to result in a gain of approximately 0.64 QALYs over those obtained with PR48 treatment (Figure 4, Table 5). This corresponds to ICERs of $15,067/QALY and $56,013/QALY for the BOC/RGT and BOC/PR48 treatment regimens compared to the PR48 regimen, respectively. In the black cohort, treatment with BOC/RGT was projected to result in a gain of 0.47 QALYs and treatment with BOC/PR48 was projected to result in a gain of 0.68 QALYs over those obtained with PR48 treatment. This corresponds to ICERs of $30,627/QALY and $50,423/QALY for the BOC/RGT and BOC/PR48 treatment regimens, respectively, when compared to treatment with PR48. The ICER of BOC/PR48 compared with BOC/RGT in treating black patients is $94,610/QALY.

The analysis comparing the boceprevir label recommendation relative to dual therapy resulted in an average increase of $18,047 in cost and 0.66 in QALYs and the corresponding ICER was $27,265/QALY (Table 5).

**Table 5:** Change in total discounted lifetime costs (2010 US$) and quality adjusted life years of boceprevir-based regimens compared with PR48 in multi-way sensitivity and subset analyses

	**BOC/RGT vs. PR48**			**BOC/PR48 vs. PR48**		
	**Discounted costs**	**Discounted QALYs**	**ICER**	**Discounted costs**	**Discounted QALYs**	**ICER**
**Base**	10,348	0.62	16,792	35,727	0.65	55,162
**Sensitivity Analyses**						
Discount rate for costs = 0%, discount rate for utilities = 0%	2,868	1.23	2,338	27,870	1.33	21,016
Discount rate for costs = 5%, discount rate for utilities = 5%	12,894	0.42	30,630	38,256	0.43	88,789
Lower Bound of Transition Rates	12,126	0.43	28,314	37,703	0.44	85,867
Upper Bound of Transition Rates	11,032	0.83	13,340	36,485	0.88	41,393
Lower Bound for Health State Costs	12,538	0.62	20,346	38,165	0.65	58,927
Upper Bound for Health State Costs	8,158	0.62	13,239	33,288	0.65	51,397
Lower Bound for Population Average Utilities	10,348	0.61	17,097	35,727	0.64	56,228
Upper Bound for Population Average Utilities	10,348	0.63	16,508	35,727	0.66	54,133
Lower Bound for On Treatment Utilities	10,348	0.62	16,724	35,727	0.64	56,120
Upper Bound for On Treatment Utilities	10,348	0.62	16,819	35,727	0.66	53,800
Lower Bound for Utilities of SVR states	10,348	0.31	33,511	35,727	0.30	117,395
Upper Bound for Utilities of SVR states	10,348	0.62	16,792	35,727	0.65	55,162
Lower Bound for Health State Utilities	10,348	0.95	10,906	35,727	1.02	34,927
Upper Bound for Health State Utilities	10,348	0.33	31,124	35,727	0.33	108,965
**Subset Analyses**						
Non-Black Cohort	9,655	0.64	15,067	35,968	0.64	56,013
Black Cohort	14,437	0.47	30,627	34,305	0.68	50,423
	**BOC regimen vs. PR48**					
	**Discounted Costs**	**Discounted Utilities**	**ICER**			
Label-Based Analyses	18,046	0.66	27,265			

PR48 – peginterferon-ribavirin regimen for 48 weeks; BOC/RGT – peginterferon–ribavirin for 4 weeks followed by peginterferon-ribavirin and boceprevir for 24 weeks, and those with a detectable hepatitis C virus (HCV) RNA level between weeks 8 and 24 received peginterferon–ribavirin from week 28 to week 48; BOC/PR48 –peginterferon–ribavirin for 48 weeks and boceprevir for 44 weeks.

## Discussion

In our model, we assumed that SVR is a cure for mild and moderate HCV and that patients who achieve an SVR through AV therapy will not be at risk for developing serious and costly complications associated with HCV. There are a small number of studies which suggest that patients with moderate HCV may develop HCC even after achieving an SVR with drug therapy [37,38]. The limited data suggests that the probability of this transition is very close to zero. Because of the limited information and since the transition is negligible, we did not include it in our model. Data also suggests that cirrhotic patients may have a regression of fibrosis if they achieve an SVR, which would lower their risk of developing HCV-related liver complications. A recently published study by van der Meer et al. [58] showed that the all-cause mortality rates in patients who achieved SVR and who did not achieve SVR were 8.9% and 27% at 10 years, respectively. They also reported that 10-year cumulative incidence rates of HCC and decompensated cirrhosis in patients who achieved SVR were 5.1% and 2.1%, respectively. In our analysis, we also included a progression of disease in cirrhotic patients who achieved SVR, and the incidence rates reported by van der Meer et al. were included in our sensitivity analysis range.

SPRINT-2 demonstrated that the addition of boceprevir to peginterferon alfa-2b and ribavirin after a 4-week peginterferon─ribavirin lead-in period significantly increased SVR rate over treatment with peginterferon─ribavirin alone in previously untreated adult patients infected with HCV genotype 1. However, the boceprevir-based treatment regimens themselves are more costly than treatment with peginterferon and ribavirin alone. Given the scarce resources and competing demands, payers often need to consider the long-term impact that treatment will have on the clinical and economic burden of disease. Our modeling study assessed the cost-effectiveness of the boceprevir-based strategies studied in SPRINT-2 over the lifetime of patients from the payer perspective. We also examined the impact of the FDA-approved label-based strategies on the incidence of HCV-related complications, lifetime costs, QALYS, and assessed the cost-effectiveness of these regimens.

Our model estimates that treatment with PR48 is associated with considerable reductions on the incidence of serious liver complications compared to no treatment and is cost-effective at commonly used thresholds. However, our model projections indicate that treatment with boceprevir-based regimens offer substantial additional benefit.

A difference in the incidence rates of serious liver disease between boceprevir-based regimens and PR48 was projected to occur within 10 years following treatment (Figure 2). Hence, although the AV therapy costs of BOC/RGT and BOC/PR48 are considerably greater than the AV therapy costs of PR48, a savings in the projected costs of managing HCV-related liver disease is expected to offset some of the drug costs. The ICERs of the BOC/RGT and BOC/PR48 treatment regimens compared with PR48 were $16,792/QALY and $55,162/QALY, respectively. Thus both BOC/RGT and BOC/PR48 are considered cost-effective at commonly used thresholds [59]. In addition, the ICER of BOC/PR48 compared with BOC/RGT was $807,804/QALY, which implies that BOC/PR48 is not cost-effective at commonly used thresholds when compared to treatment with BOC/RGT. The high ICER obtained from comparing BOC/PR48 to BOC/RGT is mostly explained by the small difference in SVR rates between the two treatment strategies (BOC/PR48: 66% vs. BOC/RGT: 63%) and the difference in AV therapy costs (BOC/PR48:$69,928 vs. BOC/RGT: $47,582).

Describing the natural history of chronic HCV has been historically difficult because acute infection is often asymptomatic, and the duration between infection and development of advanced stages of liver disease is typically long [60]. Because of the variability of the estimates reported in literature and potential variability in treatment efficacy, we conducted sensitivity analyses on the majority of model inputs – treatment efficacy, transition rates, health state costs, and the quality of life associated with the health states. Results on the costs, benefits and cost-effectiveness of treatment varied widely across the different scenarios considered. The majority of one-way sensitivity analyses did not substantially impact the ICERs compared to the base case analyses. Only 5 of the 103 scenarios evaluated resulted in an ICER comparing BOC/RGT to PR48 that was more than $5000 different from the base case analysis ($16,792/QALY). Specifically, assumptions regarding the utility of the SVR-F1 health state, efficacy of PR48 and BOC/RGT, and the discount rates were most impactful on the ICER. Similarly, only 18 of the 103 scenarios evaluated resulted in an ICER comparing BOC/PR48 to PR48 that was more than $5,000 different from the base case analysis ($55,162/QALY). Specifically, assumptions regarding the transition rates from F4 to DC and F4 to HCC; utility of the F1-F4, SVR-F1, SVR-F2 health states; efficacy of PR48 and BOC/PR48; and the discount rates were most impactful on the ICER. These results imply that in comparison to treatment with dual therapy, the ICERs of BOC/RGT and BOC/PR48 are robust if a single model parameter is changed.

Multivariate sensitivity analyses found that there is more variability in the cost-effectiveness ratios associated with BOC/PR48 vs. PR48 treatment than in the cost-effectiveness ratios associated with BOC/RGT vs. PR48. The ICERs were most sensitive to assumptions concerning the quality of life of the HCV health states and least sensitive to assumptions concerning the quality of life of patients on treatment for those who receive BOC/RGT, and quality of life of the general population for patients who receive BOC/PR48. The most favorable results for BOC/RGT and BOC/PR48 were generated when a discount rate of 0% was applied to both costs and utilities, whereas the least favorable results were generated when the lower bounds of the SVR health states were assumed.

The PSA allowed us to evaluate the impact of varying the values of several parameters simultaneously on the projected long-term costs and QALYs of each treatment strategy at a variety of thresholds. Compared with PR48, BOC/RGT was cost-effective in nearly 100% of the 10,000 simulations when a threshold of $50,000 was chosen. This suggests that BOC/RGT offers the opportunity for a shorter duration of treatment than dual therapy that is significantly more efficacious and cost-effective at a threshold of $50,000 under a variety of assumptions.

Because of the differential treatment efficacy of dual therapy reported in non-black and black patients, data for these cohorts were collected and analyzed separately in the SPRINT-2 efficacy analyses. Although the reported SVR rates differed between the cohorts, the results of our cost-effectiveness study indicated similar trends within the results of the two subgroups. For both subgroup analyses by race cohort, compared to treatment with PR48, the ICER corresponding to the BOC/RGT treatment strategy was better than the ICER corresponding to the BOC/PR48 treatment strategy. The ICERs corresponding to BOC/PR48 compared with PR48 for both race cohorts were similar - $50,423 and $56,013 for the non-black and black cohorts, respectively. There was a greater difference in the ICERs between the boceprevir-based treatment strategies compared to dual therapy for the non-black subgroup than in the ICERs in the black subgroup. This is because the efficacies between the two boceprevir-based regimens are very similar even though the treatment cost of BOC/PR48 is much greater than the treatment cost of BOC/RGT. This implies that a longer duration of treatment may not result in additional clinical benefits. Conversely, treatment with BOC/PR48 resulted in an incremental gain of 0.21 QALYs compared to treatment with BOC/RGT in the black population. This implies that a longer duration of treatment with boceprevir may result in additional clinical benefit for black patients as is supported by the cost-effectiveness frontier (Figure 4).

The treatment strategies recommended in the FDA-approved boceprevir label include futility rules consistent with the guidelines of AASLD [21]. The ICER for the label-based treatment recommendation was $27,265 which implies that the label-based treatment recommendation is cost-effective at a reasonable threshold when compared with dual therapy. This indicates that the boceprevir-based treatment strategy offers great clinical benefit for the cost that is incurred.

Compared to previously published cost-effectiveness models [22-26], our modeling study made several updates in the model structure and inputs. First, we incorporated treatment strategies that include boceprevir – a recently approved protease inhibitor which offers the opportunity for a shorter duration of therapy and significantly greater chance of attaining a cure. In addition, we updated the transition probabilities associated with progression of HCV, development of serious liver disease, the probability of receiving a liver transplant, and health state costs using data that was not previously available. Finally, unlike the majority of previous models, we included treatment of patients with cirrhosis in our model. We assumed that cirrhotic patients achieved a partial cure from HCV even if they attained SVR with treatment. This feature of our model –that patients with cirrhosis who achieve SVR are at risk of developing decompensated cirrhosis and hepatocellular carcinoma – was not incorporated in the majority of previous models.

After we developed our model, Liu et al. published a cost-effectiveness modeling study that included the costs and efficacy of recently approved protease inhibitors in the treatment regimen [27]. Our model differs from this modeling study in several ways. Specifically, the transition probabilities and health state costs included in our model are based on more recent data than those applied in the Liu model. Unlike Liu et al. which included cost for HCV with SVR and out-of-pocket expenses as part of the base case scenario, we only tested the influence of cost for HCV with SVR in the sensitivity analysis and we did not consider out-of-pocket expenses. Although the Liu model includes treatment of patients with cirrhosis, it was assumed that patients with cirrhosis who achieved an SVR were permanently cured of HCV. We assumed that patients with cirrhosis achieved a partial cure from HCV even if they attained SVR with treatment, which is consistent with recently published data [28]. Finally, we also evaluated the treatment regimen recommended by the FDA and AASLD for boceprevir, not just the treatment regimens studied in SPRINT-2.

Our study has several limitations. First, we did not model the possibility of retreatment with antiviral drugs that are currently available or might become available in the near future. Assumptions could be made concerning the timing of re-treatment in patients who received dual therapy in SPRINT-2 using data collected from a trial in previously treated patients (RESPOND-2) [18]. However, the efficacy of re-treatment for patients who did not achieve an SVR with one of the boceprevir-based regimens is unknown. Second, our model did not take into consideration the possibility of re-infection nor re-transplantation in patients who receive a liver transplant. These patients are at increased risk for developing future liver complications and receiving subsequent liver transplants. Since re-transplantation would occur further in the future, its influence on costs and benefits would be heavily discounted. This suggests that the inclusion of re-transplantation would have a favorable but small impact on the cost-effectiveness of boceprevir and that the current modeling study provides a conservative estimate of the cost-effectiveness of boceprevir-based regimens. Third, our model cannot be applied to special populations such as patients co-infected with HIV because of lack of data on the impact of treatment with boceprevir-based regimens at this time. Fourth, our study applies the all-cause mortality rate to patients with chronic HCV and to patients who attain an SVR. Subsets of this patient population are considered high-risk and the mortality rate of the general population may underestimate the mortality rate of the HCV treatment population. Our model also does not take into account that patients who attain an SVR are at risk for reinfection with HCV. This assumption may bias the results in favor of boceprevir-based regimens since the boceprevir-based regimens reported higher SVR rates. Sixth, our model does not take into account that patients who do not achieve an SVR with AV therapy may receive some benefit, such as a slower disease progression rate. The impact of relaxing this assumption on the results is not clear a priori. Seventh, this analysis was done from the payer perspective. Patients with chronic HCV or the sequelae of cirrhosis have been shown to experience increased work and productivity losses, suffer activity impairment, and incur increased indirect medical costs compared with people without HCV [61-63]. Inclusion of such costs would result in lower ICERs for both boceprevir-regimens compared with dual therapy since the treatment efficacy of both BOC/RGT and BOC/PR48 are greater than the efficacy of treatment with PR48. Finally, all treatment characteristics are based entirely on clinical trial data. The discontinuation rates, which impact treatment efficacy, may differ in clinical practice.

## Conclusion

In summary, boceprevir-based regimens were projected to substantially reduce the burden of liver-related complications such as decompensated cirrhosis, hepatocellular carcinoma, liver-related mortality, and liver-transplants in treatment-naïve patients infected with hepatitis C genotype 1. Our model also demonstrated that boceprevir-based regimens offer patients the possibility of experiencing great clinical benefit with a shorter duration of therapy that may minimize the time patients experience an HCV-treatment decrement to their quality of life. In addition both BOC/RGT and BOC/PR48 were projected to be cost-effective from the payer perspective at a reasonable threshold in comparison with treatment with peginterferon and ribavirin alone.

## Abbreviations

AASLD: American Association for the Study of Liver Diseases; AV: Antiviral; BOC: Boceprevir; BOC/PR48: Fixed duration of therapy for BOC, peginterferon and ribavirin for 4 weeks followed by BOC plus peginterferon and ribavirin for 44 weeks; BOC: Plus peginterferon and ribavirin for 44 weeks; BOC/RGT: Response-guided therapy; DC: Decompensated cirrhosis; EPO: Erythropoietin; ETR: End of treatment response; F0: No fibrosis; F1: Portal fibrosis without septa; F2: Portal fibrosis with few septa; F3: Numerous septa without cirrhosis; F4: Cirrhosis; FDA: Food and Drug Administration; HCV: Hepatitis c virus; HCC: Hepatocellular carcinoma; ICER: Incremental cost-effectiveness ratio; Lv-death: Liver-related death; LT: Liver transplantation; N: No; P: Peginterferon; PLT: Post-liver transplantation; PR48: peginterferon and ribavirin for 4 weeks followed by placebo plus peginterferon and ribavirin for 44 weeks; QALY: Quality-adjusted life years; R: Ribavirin; SPRINT-2: Serine protease inhibitor therapy 2; SRTR: Scientific Registry of Liver Transplant Recipients; SVR: Sustained virologic response; Tx: Treatment; US: United States; Y: Yes

## Competing interests

This study was sponsored by Schering-Plough (now part of Merck Sharp & Dohme Corp., a subsidiary of Merck & Co., Inc., Whitehouse Station, NJ). All authors have completed the ICMJE Form for Disclosure of Potential Conflicts of Interest and report the following: Drs. Ferrante, El Khoury, and Elbasha are current employees of Merck Sharp & Dohme Corp., a subsidiary of Merck & Co., Inc., Whitehouse Station, NJ, and hold stock and/or stock options. Dr. Chhatwal is a former employee of Merck and has received consulting fees. Dr. Brass is a former employee of Merck and holds stock and/or stock options. Dr Poordad has received consultancy fees from Merck, Vertex, Abbott, Gilead, Achillion, Genentech, and Tibotec; has grants/grants pending from Merck; and has received payment for development of educational presentations and speaker fees from Merck, Genentech, Salix and Gilead. Dr Bronowicki has received consultancy fees from Schering-Plough (now part of Merck), Roche, Gilead, Bristol Myers Squibb, Janssen, Boehringer Ingelheim, Novartis, and Bayer; payment for lectures including service on speakers bureaus for Schering-Plough (now part of Merck), Roche, Bayer and Bristol Myers Squibb and travel/accommodations/meeting expenses unrelated to activities listed from Roche. The corresponding author had full access to all of the data and takes full responsibility for the veracity of the data and statistical analysis.

## Authors’ contributions

CAB, designed the original SPRINT-2 study. SAF, JC, ACEK, and EHE developed the model. FP and JPB enrolled patients and/or contributed to data collection for the original study. All authors provided critical input to the draft. All authors reviewed the final draft and agree with its content.

## Pre-publication history

The pre-publication history for this paper can be accessed here:

http://www.biomedcentral.com/1471-2334/13/190/prepub

## Supplementary Material

Additional file 1: Table S1 List of protocol and center number and the responsible institutional review board. Click here for file

Additional file 2: Table S2 Summary of Costs, QALYs, and ICERs of One-Way Sensitivity Analyses. BOC/RGT vs PR48 and BOC/PR48 vs. PR48. Click here for file

## References

[B1] World Health Organization (WHO) Hepatitis: facts and figures 2010 http://www.euro.who.int/en/what-we-do/health-topics/communicable-diseases/hepatitis/facts-and-figures/hepatitis-c (accessed September 9, 2011)

[B2] Amarapurkar D Natural history of hepatitis C virus infection J Gastroenterol Hepatol 2000 15 suppl E105 E110 10.1046/j.1440-1746.2000.02110.x 10921391

[B3] Di Bisceglie AM Natural history of hepatitis C: its impact on clinical management Hepatol 2000 31 1014 1018 10.1053/he.2000.5762 10733560

[B4] Annual Report of the U.S. Organ Procurement and Transplantation Network and the Scientific Registry of Transplant Recipients Transplant Data 1999–2008 2009 Rockville, MD: U.S. Department of Health and Human Services, Health Resources and Services Administration, Healthcare Systems Bureau, Division of Transplantation

[B5] Te HS Jensen DM Epidemiology of hepatitis B and C viruses: a global overview Clin Liver Dis 2010 14 1 1 21 vii 10.1016/j.cld.2009.11.009 20123436

[B6] Armstrong GL Wasley A Simard EP McQuillan GM Kuhnert WL Alter MJ The prevalence of hepatitis C virus infection in the United States, 1999 through 2002 Ann Intern Med 2006 144 705 714 16702586 10.7326/0003-4819-144-10-200605160-00004

[B7] El Khoury AC Klimack WK Wallace C Razavi H Economic Burden of Hepatitic C Associated Diseases in the United States J Viral Hepat 2012 19 3 153 160 10.1111/j.1365-2893.2011.01563.x 22329369

[B8] Ly KN Xing J Klevens RM Jiles RB Ward JW Holmberg SD The increasing burden of mortality from viral hepatitis in the United States between 1999 and 2007 Ann Intern Med 2012 156 4 271 278 22351712 10.7326/0003-4819-156-4-201202210-00004

[B9] Razavi H El Khoury A Elbasha E Estes C Pasini K Poynard T Kumar R Chronic Hepatitis C Virus (Hcv) Disease Burden and Cost in the United States Hepatol 2012 [Epub ahead of print] 10.1002/hep.26218 PMC3763475 23280550

[B10] Davis GL Albright JE Cook SF Rosenberg DM **Projecting future complications of chronic hepatitis C in the United States** Liver Transpl 2003 9 4 331 338 10.1053/jlts.2003.50073 12682882

[B11] Simmonds P Bukh J Combet C Deleage G Enomoto N Feinstone S Halfon P Inchauspe G Kuiken C Maertens G Mizokami M Murphy DG Okamoto H Pawlotsky JM Penin F Sablon E Shin-I T Stuyver LJ Thiel HJ Viazov S Weiner AJ Widell A Consensus proposals for a unified system of nomenclature of hepatitis C virus genotypes Hepatol 2005 42 962 973 10.1002/hep.20819 16149085

[B12] Wilkins T Malcolm JK Raina D Schade RR Hepatitis C: diagnosis and treatment Am Fam Physician 2010 81 11 1351 1357 20521755

[B13] Ghany MG Strader DB Thomas DL Seeff LB Diagnosis, management, and treatment of hepatitis C: an update Hepatol 2009 49 4 1335 1374 10.1002/hep.22759 PMC7477893 19330875

[B14] Manns MP McHutchison JG Gordon SC Rustgi VK Shiffman M Reindollar R Goodman ZD Koury K Ling M Albrecht JK Peginterferon alfa–2.b plus ribavirin compared with interferon alfa–2b plus ribavirin for initial treatment of chronic hepatitis C: a randomised trial Lancet 2001 358 958 965 10.1016/S0140-6736(01)06102-5 11583749

[B15] Fried MW Shiffman ML Reddy KR Smith C Marinos G Gonçales FL Jr Häussinger D Diago M Carosi G Dhumeaux D Craxi A Lin A Hoffman J Yu J Peginterferon alfa–2a plus ribavirin for chronic hepatitis C virus infection N Engl J Med 2002 347 975 982 10.1056/NEJMoa020047 12324553

[B16] Muir AJ Bornstein JD Killenberg PG Atlantic Coast Hepatitis Treatment Group Peginterferon alfa–2b and ribavirin for the treatment of chronic hepatitis C in blacks and non–Hispanic whites N Engl J Med 2004 350 2265 2271 Erratum, N Engl J Med 2004;351:1268 10.1056/NEJMoa032502 15163776

[B17] Poordad F McCone J Bacon BR Bruno S Manns MP Sulkowski MS Jacobson IM Reddy KR Goodman ZD Boparai N DiNubile MJ Sniukiene V Brass CA Albrecht JK Bronowicki JP SPRINT-2 Investigators Boceprevir for untreated chronic HCV genotype 1 infection N Engl J Med 2011 364 13 1195 1206 10.1056/NEJMoa1010494 21449783 PMC3766849

[B18] Bacon BR Gordon SC Lawitz E Marcellin P Vierling JM Zeuzem S Poordad F Goodman ZD Sings HL Boparai N Burroughs M Brass CA Albrecht JK Esteban R HCV RESPOND-2 Investigators Boceprevir for previously treated chronic HCV genotype 1 infection N Engl J Med 2011 364 1207 1217 10.1056/NEJMoa1009482 21449784 PMC3153125

[B19] Jacobson IM McHutchison JG Dusheiko G Di Bisceglie AM Reddy KR Bzowej NH Marcellin P Muir AJ Ferenci P Flisiak R George J Rizzetto M Shouval D Sola R Terg RA Yoshida EM Adda N Bengtsson L Sankoh AJ Kieffer TL George S Kauffman RS Zeuzem S ADVANCE Study Team Telaprevir for previously untreated chronic hepatitis C virus infection N Engl J Med 2011 364 2407 2416 10.1056/NEJMoa1012912 21696307

[B20] Zeuzem S Andreone P Pol S Lawitz E Diago M Roberts S Focaccia R Younossi Z Foster GR Horban A Ferenci P Nevens F Müllhaupt B Pockros P Terg R Shouval D van Hoek B Weiland O Van Heeswijk R De Meyer S Luo D Boogaerts G Polo R Picchio G Beumont M REALIZE Study Team Telaprevir for retreatment of HCV infection N Engl J Med 2011 364 2417 2428 10.1056/NEJMoa1013086 21696308

[B21] Ghany MG Nelson DR Strader DB Thomas DL Seeff LB An update on treatment of genotype 1 chronic hepatitis C virus infection: 2011 practice guideline by the American Association for the study of liver diseases Hepatol 2011 54 4 1433 1444 10.1002/hep.24641 PMC3229841 21898493

[B22] Buti M Medina M Casado MA Wong JB A cost–effectiveness analysis of peginterferon alfa–2b plus ribavirin for the treatment of naive patients with chronic hepatitis C Aliment Pharmacol Ther 2003 17 5 687 694 10.1046/j.1365-2036.2003.01453.x 12641518

[B23] Salomon JA Weinstein MC Hammitt JK Goldie SJ Cost–effectiveness of treatment for chronic hepatitis C infection in an evolving patient population JAMA 2003 290 2 228 237 10.1001/jama.290.2.228 12851278

[B24] Siebert U Sroczynski G Rossol S Wasem J Ravens-Sieberer U Kurth BM Manns MP McHutchison JG Wong JB German Hepatitis C Model (GEHMO) Group; International Hepatitis Interventional Therapy (IHIT) Group Cost effectiveness of peginterferon –2b plus ribavirin versus interferon –2b plus ribavirin for initial treatment of chronic hepatitis C Gut 2003 52 3 425 432 10.1136/gut.52.3.425 12584228 PMC1773554

[B25] Sullivan SD A Alberti A Giuliani G Giuliani G De Carli C Wintfeld N Patel KK Green J Cost effectiveness of peginterferon alpha–2a plus ribavirin versus interferon alpha–2b plus ribavirin as initial therapy for treatment–naive chronic hepatitis C Pharmacoeconomics 2004 22 4 257 265 10.2165/00019053-200422040-00004 14974875

[B26] Younossi Z Singer ME McHutchison JG Shermock KM Cost effectiveness of interferon 2b combined with ribavirin for the treatment of chronic hepatitis C Hepatol 1999 30 5 1318 1324 10.1002/hep.510300518 10534357

[B27] Liu S Cipriano LE Holodniy M Owens DK Goldhaber-Fiebert JD New protease inhibitors for the treatment of chronic hepatitis C a cost-effectiveness analysis Ann Intern Med 2012 156 279 290 22351713 10.1059/0003-4819-156-4-201202210-00005 PMC3586733

[B28] Cardoso AC Moucar R Figueiredo-Mendes C Ripault MP Giuily N Castelnau C Boyer N Asselah T Martinot-Peignoux M Maylin S Carvalho-Filho RJ Valla D Bedossa P Marcellin P Impact of peginterferon and ribavirin therapy on hepatocellular carcinoma: incidence and survival in hepatitis C patients with advanced fibrosis J Hepatol 2010 52 5 652 657 10.1016/j.jhep.2009.12.028 20346533

[B29] Thein HH Yi Q Dore GJ Krahn MD Estimation of stage-specific fibrosis progression rates in chronic hepatitis C virus infection: A meta-analysis and meta-regression Hepatol 2008 48 2 418 431 10.1002/hep.22375 18563841

[B30] Fattovich G Giustina G Degos F Tremolada F Diodati G Almasio P Nevens F Solinas A Mura D Brouwer JT Thomas H Njapoum C Casarin C Bonetti P Fuschi P Basho J Tocco A Bhalla A Galassini R Noventa F Schalm SW Realdi G Morbidity and mortality in compensated cirrhosis type C: A retrospective follow-up study of 384 patients Gastroenterol 1997 112 2 463 472 10.1053/gast.1997.v112.pm9024300 9024300

[B31] Gentilini P Laffi G La Villa G Romanelli RG Buzzelli G Casini-Raggi V Melani L Mazzanti R Riccardi D Pinzani M Zignego AL Long course and prognostic factors of virus-induced cirrhosis of the liver Am J Gastroenterol 1997 92 1 66 72 8995940

[B32] Serfaty L Chazouillères O Bonnand AM Bonnand AM Rosmorduc O Poupon RE Poupon R Determinants of outcome of compensated hepatitis C virus-related cirrhosis Hepatol 1998 27 5 1435 1440 10.1002/hep.510270535 9581703

[B33] Benvegnu L Gios M Boccato S Alberti A Natural history of compensated viral cirrhosis: a prospective study on the incidence and hierarchy of major complications Gut 2004 53 744 749 10.1136/gut.2003.020263 15082595 PMC1774055

[B34] Sangiovanni A Prati GM Fasani P Ronchi G Romeo R Manini M Del Ninno E Morabito A Colombo M The natural history of compensated cirrhosis due to hepatitis c virus: a 17-year cohort study of 214 patients Viral Hepat 2006 43 6 1303 1310 10.1002/hep.21176 16729298

[B35] Tsukuma H Hiyama T Tanaka S Nakao M Yabuuchi T Kitamura T Nakanishi K Fujimoto I Inoue A Yamazaki H Risk factors for hepatocellular carcinoma among patients with chronic liver disease N Engl J Med 1993 328 25 1797 1801 10.1056/NEJM199306243282501 7684822

[B36] Bruno S Silini E Crosignani A Borzio F Leandro G Bono F Asti M Rossi S Larghi A Cerino A Podda M Mondelli MU Hepatitis c virus genotypes and risk of hepatocellular carcinoma in cirrhosis: a prospective study Hepatol 1997 25 3 754 758 10.1002/hep.510250344 9049231

[B37] Yoshida H Shiratori Y Moriyama M Arakawa Y Ide T Sata M Inoue O Yano M Tanaka M Fujiyama S Nishiguchi S Kuroki T Imazeki F Yokosuka O Kinoyama S Yamada G Omata M Interferon therapy reduces the risk for hepatocellular carcinoma: national surveillance program of cirrhotic and noncirrhotic patients with chronic hepatitis c in japan: Inhibition of hepatocarcinogenesis by interferon therapy Ann Intern Med 1999 131 3 174 181 10428733 10.7326/0003-4819-131-3-199908030-00003

[B38] Tateyama M Yatsuhashi H Taura N Motoyoshi Y Nagaoka S Yanagi K Abiru S Yano K Komori A Migita K Nakamura M Nagahama H Sasaki Y Miyakawa Y Ishibashi H Alpha-fetoprotein above normal levels as a risk factor for the development of hepatocellular carcinoma in patients infected with hepatitis c virus J Gastroenterol 2011 46 1 92 100 10.1007/s00535-010-0293-6 20711614

[B39] Planas R Ballesté B Alvarez MA Rivera M Montoliu S Galeras JA Santos J Coll S Morillas RM Solà R Natural history of decompensated hepatitis c virus-related cirrhosis: A study of 200 patients J Hepatol 2004 40 5 823 830 10.1016/j.jhep.2004.01.005 15094231

[B40] UNOS and the Division of Organ Transplantation, Health Resources and Service Administration Center Specific Report: liver data, UNOS Update 1995 1994 11 15–18

[B41] Thuluvath PJ Guidinger MK Fung JJ Johnson LB Rayhill SC Pelletier SJ Liver Transplantation in the united states, 1999–2008 Am J Transpl 2010 10 4 89 99 10.1111/j.1600-6143.2010.03037.x 20420649

[B42] Davis GL Alter MJ El–Serag H Poynard T Jennings LW Aging of hepatitis C virus (HCV)-infected persons in the United States: a multiple cohort model of HCV prevalence and disease progression Gastroenterol 2010 138 2 513 521 10.1053/j.gastro.2009.09.067 19861128

[B43] Lang K Danchenko N Gondek K Shah S Thompson D The burden of illness associated with hepatocellular carcinoma in the United States J Hepatol 2009 50 1 89 99 10.1016/j.jhep.2008.07.029 18977551

[B44] Arias E United states life tables, 2006: National vital statistics reports: from the Centers for Disease Control and Prevention, National Center for Health Statistics Natl Vital Stat Syst 2010 58 1 21043319

[B45] Wolfe RA Merion RM Roys EC Port FK Trends in Organ Donation and Transplantation in the United States, 1999–2008 Am J Transplant 2010 10 4p2 961 972 10.1111/j.1600-6143.2010.03021.x 20420646

[B46] First DataBank, Inc Drug databases [http://www.firstdatabank.com/Support/drug-pricing-policy.aspz]

[B47] Wilson J Yao G Raftery J Bohlius J Brunskill S Sandercock J Bayliss S Moss P Stanworth S Hyde C A systematic review and economic evaluation of epoetin alpha, epoetin beta and darbepoetin alpha in anaemia associated with cancer, especially that attributable to cancer treatment Health Technol Assess 2007 11 13 1 202 iii–iv 17408534 10.3310/hta11130

[B48] Hanmer J Lawrence WF Anderson JP Kaplan RM Fryback DG Report of nationally representative values for the noninstitutionalized US adult population for 7 health-related quality-of-life scores Med Decis Making 2006 26 391 400 10.1177/0272989X06290497 16855127

[B49] McAdam-Marx C McGarry LJ Hane CA Biskupiak J Deniz B Brixner DI All-cause and incremental per patient per year cost associated with chronic hepatitis C virus and associated liver complications in the United States: a managed care perspective J Manag Care 2011 17 531 546 10.18553/jmcp.2011.17.7.531 PMC10438304 21870894

[B50] Davis KL Mitra D Medjedovic J Beam C Rustgi V Direct economic burden of chronic hepatitis C virus in a United States managed care population J Clin Gastroenterol 2011 45 e17 24 10.1097/MCG.0b013e3181e12c09 20628308

[B51] Chong CAKY Gulamhussein A Heathcote EJ Lilly L Sherman M Naglie G Krahn M Health-state utilities and quality of life in hepatitis C patients Am J Gastroenterol 2003 98 630 638 10.1111/j.1572-0241.2003.07332.x 12650799

[B52] Tong M El-Farra NS Reikes AR Co RL Clinical outcomes after transfusion–associated hepatitis C N Engl J Med 1995 332 22 1463 1466 10.1056/NEJM199506013322202 7739682

[B53] Kenny-Walsh E Clinical outcomes after hepatitis C infection from contaminated anti-D immune globulin N Engl J Med 1999 340 16 1228 1233 10.1056/NEJM199904223401602 10210705

[B54] Bennet WG Inoue Y Beck R Wong JB Pauker SG Davis GL Estimates of the Cost-Effectiveness of a Single Course of Interferon-a2b in Patients with Histologically Mild Chronic Hepatitis C Ann Intern Med 1997 127 10 855 865 9382363 10.7326/0003-4819-127-10-199711150-00001

[B55] Gines P Quintero E Arroyo V Terés J Bruguera M Rimola A Caballería J Rodés J Rozman C Compensated cirrhosis – natural-history and prognostic factors Hepatol 1987 7 1 122 128 10.1002/hep.1840070124 3804191

[B56] Manns MP Wedemeyer H Cornberg M Treating viral hepatitis C: efficacy, side effects, and complications Gut 2006 55 9 1350 1359 10.1136/gut.2005.076646 16905701 PMC1860034

[B57] Victrelis prescribing information[http://www.merck.com/product/usa/pi_circulars/v/victrelis/victrelis_pi.pdf] Accessed March 21, 2012

[B58] van der Meer AJ Veldt BJ Feld JJ Wedemeyer H Dufour JF Lammert F Duarte-Rojo A Heathcote EJ Manns MP Kuske L Zeuzem S Hofmann WP de Knegt RJ Hansen BE Janssen HL Association between sustained virological response and all-cause mortality among patients with chronic hepatitis C and advanced hepatic fibrosis JAMA 2012 308 24 2584 2593 10.1001/jama.2012.144878 23268517

[B59] Owens DK Interpretation of cost-effectiveness analyses J Gen Intern Med 1998 13 10 716 717 10.1046/j.1525-1497.1998.00211.x 9798822 PMC1497852

[B60] Seeff LB Buskell-Bales Z Wright EC Durako SJ Alter HJ Iber FL Hollinger FB Gitnick G Knodell RG Perrillo RP Long-term mortality after transfusion-associated non-a, non-B hepatitis: The National Heart, Lung, and Blood Institute Study Group N Engl J Med 1992 327 1906 1911 10.1056/NEJM199212313272703 1454085

[B61] Su J Brook RA Kleinman NL Corey-Lisle P The impact of hepatitis C virus infection on work absence, productivity, and healthcare benefit costs Hepatol 2010 52 2 436 442 10.1002/hep.23726 20683943

[B62] DiBonaventura M Wagner JS Yuan Y L’Italien G Langley P Ray Kim W The impact of hepatitis C on labor force participation, absenteeism, presenteeism and non-work activities J Med Econ 2011 14 2 253 261 10.3111/13696998.2011.566294 21385147

[B63] El Khoury AC Vietri J Prajapati G The burden of untreated hepatitis C virus infection: a US patients’ perspective Dig Dis Sci 2012 57 11 2995 3003 10.1007/s10620-012-2233-1 22674399

