# Supplementary material for: Boceprevir for previously untreated patients with chronic hepatitis C Genotype 1 infection: a US-based cost-effectiveness modeling study
Source: BMC Infect Dis. 2013 Apr 27;13:190. doi: 10.1186/1471-2334-13-190 (PMC3643851; doi:10.1186/1471-2334-13-190)
Supplement: Additional file 1: Table S1 — List of protocol and center number and the responsible institutional review board. [file 1471-2334-13-190-S1.pdf]

Protocol and Center Number	Responsible IRB Name and Address(es)
P05216-01	CPP "Sud-Est II"<br>Hopital Hotel Dieu<br>1 Place de l'Hopital, Porte 16<br>Lyon Cedex 02 69288<br>France
P05216-02	CPP "Sud-Est II"<br>Hopital Hotel Dieu<br>1 Place de l'Hopital, Porte 16<br>Lyon Cedex 02 69288<br>France
P05216-03	CPP "Sud-Est II"<br>Hopital Hotel Dieu<br>1 Place de l'Hopital, Porte 16<br>Lyon Cedex 02 69288<br>France
P05216-04	CPP "Sud-Est II",<br>Hopital Hotel Dieu<br>1 Place de l'Hopital, Porte 16<br>Lyon Cedex 02 69288<br>France
P05216-05	CPP "Sud-Est II"<br>Hopital Hotel Dieu<br>1 Place de l'Hopital, Porte 16<br>Lyon Cedex 02, 69288<br>France
P05216-06	CPP "Sud-Est II"<br>Hopital Hotel Dieu<br>1 Place de l'Hopital, Porte 16<br>Lyon Cedex 02, 69288<br>France
P05216-07	CPP "Sud-Est II"<br>Hopital Hotel Dieu<br>1 Place de l'Hopital, Porte 16<br>Lyon Cedex 02, 69288

Protocol and Center Number	Responsible IRB Name and Address(es)
	France
P05216-08	CPP "Sud-Est II"<br>Hopital Hotel Dieu<br>1 Place de l'Hopital, Porte 16<br>Lyon Cedex 02, 69288<br>France
P05216-09	BIDMC Committee on Clinical Investigations<br>330 Brookline Ave, E/FN-201<br>Boston MA, 02215<br>USA
P05216-10	Saint Louis University Institutional Review Board<br>3556 Caroline Street, Room 110<br>Saint Louis MO, 63104<br>USA
P05216-11	Tulane University<br>1440 Tulane Ave. TW36 Tidewater Bldg, Ste 1705<br>Office of Human Research Protection<br>New Orleans LA, 70112-2699<br>USA
P05216-12	WIRB<br>3535 7th Ave. Southwest<br>Olympia WA, 98502 - 5010<br>USA
P05216-13	Western Institutional Review Board<br>3535 7th Avenue SouthWest<br>Olympia WA, 98502-5010<br>USA
P05216-14	University of Arizona<br>1618 East Helen Street<br>Human Subject's Committee<br>Tucson AZ, 85719<br>USA<br><br>Western Institutional Review Board<br>3535 7th Ave. SW<br>Olympia WA, 98502-5010<br>USA
P05216-18	Western Institutional Review Board<br>3535 Seventh Avenue SW<br>Olympia WA, 98502-5010<br>USA
P05216-19	Northwestern University<br>Office for Sponsored Research (OSR)<br>750 N. Lake Shore Drive<br>Rubloff 7th Floor<br>Chicago IL, 60611-3008<br>USA
P05216-20	Western Institutional Review Board<br>3535 7th Ave. SW<br>Olympia WA, 98502-5010<br>USA
P05216-21	Western Institutional Review Board<br>3535 Seventh Avenue SW<br>Olympia WA, 98502-5010<br>USA
P05216-22	Western Institutional Review Board

Protocol and Center Number	Responsible IRB Name and Address(es)
	3535 7th Ave. SW<br>Olympia WA, 98502-5010<br>USA
P05216-32	IUPUI & Clarian IRB Institution Review Boards<br>541 Clinical Drive<br>Clinical Building 365/Rm 618<br>INDIANAPOLIS IN, 46202-5111<br>USA<br><br>Research and Sponsored Programs<br>620 Union Drive, Room 618<br>Indianapolis, IN 46202-5167
P05216-33	Western Institutional Review Board<br>3535 Seventh Avenue, SW<br>Olympia WA, 98508-5010<br>USA
P05216-34	University of Texas Southwestern Medical Center<br>Institutional Review Board<br>5323 Harry Hines Blvd, Room C1.206<br>Dallas TX, 75390-8843<br>USA
P05216-35	Dean Institutional Review Board<br>2711 Allen Blvd., Suite 300<br>Middleton WI, 53562<br>USA
P05216-36	Western Institutional Board<br>3535 7th Avenue Southwest<br>Olympia WA, 98502-5010<br>USA
P05216-37	Penn State Hershey College of Med<br>Human Subj Prot. Office<br>600 Centerview Drive<br>Rm 1140, MS-A115, PO 855<br>Acad. Support Bldg<br>Hershey PA, 17033<br>USA
P05216-38	Duke University Health System<br>Institutional Review Board<br>Hock Plaza,<br>2424 Erwin Road, Suite 405<br>Campus Box 2712<br>Durham NC, 27705<br>USA
P05216-39	VA Long Beach Healthcare System<br>IRB<br>5901 East Seventh Street<br>Mail Code 09/151<br>Long Beach CA, 90822<br>USA<br><br>Institutional Review Board<br>Subcommittee on Human Subjects<br>5901 East Seventh Street

Protocol and Center Number	Responsible IRB Name and Address(es)
	3535 7th Avenue Southwest<br>OLYMPIA WA, 98502-5010<br>USA
P05216-23	Western Institutional Review Board<br>3535 Seventh Avenue SW<br>Olympia WA, 98502-5010<br>USA
P05216-24	Lancaster General Hospital Institutional Review Committee<br>555 North Duke Street<br>Lancaster PA, 17604<br>USA
P05216-25	Western Institutional Review Board<br>3535 Seventh Avenue SW<br>Olympia WA, 98502-5010<br>USA
P05216-26	Henry Ford Health System<br>2799 W. Grand Blvd. Henry Ford Hospital CFP-046<br>Institutional Review Board<br>Henry Ford Hospital CFP-046<br>Detroit MI, 48202<br>USA
P05216-27	University of South Alabama<br>IRB<br>307 University Blvd. CSAB 138<br>Mobile AL, 36688<br>USA
P05216-28	Thomas Jefferson University<br>Department of Human Subject Protection<br>1015 Chestnut Street, Suite 1100<br>Philadelphia PA, 19107<br>USA
P05216-29	Weill Cornell Medical College Institutional Review Board<br>407 East 61st Street<br>1st Floor<br>New York NY, 10065<br>USA<br><br>Weill Medical College of Cornell University<br>Committee on Human Rights in Research<br>425 E. 61st Street Suite DV-301<br>New York NY, 10065<br>USA
P05216-30	Louisiana State University Health Sciences Center<br>IRB<br>1501 Kings Hwy<br>Shreveport LA, 71103<br>USA<br><br>Louisiana State University Health Sciences Center<br>Institutional Review Board<br>2627 Linwood Ave Bldg 2<br>Shreveport LA, 71103<br>USA
P05216-31	WIRB

Protocol and Center Number	Responsible IRB Name and Address(es)
	3535 7th Ave. SW<br>Olympia WA, 98502-5010<br>USA
P05216-32	IUPUI & Clarian IRB Institution Review Boards<br>541 Clinical Drive<br>Clinical Building 365/Rm 618<br>INDIANAPOLIS IN, 46202-5111<br>USA<br><br>Research and Sponsored Programs<br>620 Union Drive, Room 618<br>Indianopolis, IN 46202-5167
P05216-33	Western Institutional Review Board<br>3535 Seventh Avenue, SW<br>Olympia WA, 98508-5010<br>USA
P05216-34	University of Texas Southwestern Medical Center Institutional Review Board<br>5323 Harry Hines Blvd, Room C1.206<br>Dallas TX, 75390-8843<br>USA
P05216-35	Dean Institutional Review Board<br>2711 Allen Blvd., Suite 300<br>Middleton WI, 53562<br>USA
P05216-36	Western Institutional Board<br>3535 7th Avenue Southwest<br>Olympia WA, 98502-5010<br>USA
P05216-37	Penn State Hershey College of Med<br>Human Subj Prot. Office<br>600 Centerview Drive<br>Rm 1140, MS-A115, PO 855<br>Acad. Support Bldg<br>Hershey PA, 17033<br>USA
P05216-38	Duke University Health System<br>Institutional Review Board<br>Hock Plaza,<br>2424 Erwin Road, Suite 405<br>Campus Box 2712<br>Durham NC, 27705<br>USA
P05216-39	VA Long Beach Healthcare System<br>IRB<br>5901 East Seventh Street<br>Mail Code 09/151<br>Long Beach CA, 90822<br>USA<br><br>Institutional Review Board<br>Subcommittee on Human Subjects<br>5901 East Seventh Street

Protocol and Center Number	Responsible IRB Name and Address(es)
	Building 162, Room 112<br>Long Beach, CA 90822
P05216-41	Western Institutional Review Board<br>3535 Seventh Avenue, SW<br>Olympia WA, 98502-5010<br>USA
P05216-42	Kaiser Permanente Southern California Institutional Review Board<br>393 East Walnut St., 2nd Floor<br>Pasadena CA, 91188<br>USA
P05216-43	Kaiser Permanente Northern IRB (KPNC IRB)<br>1800 Harris 16th Floor<br>Oakland CA, 94612<br>USA
P05216-44	Cedars-Sinai Medical Center<br>Institutional Review Board<br>Office of Research Compliance<br>8383 Wilshire Blvd., Suite 742<br>Beverly Hills CA, 90211<br>USA<br><br>Sinai Medical Center Institutional Review Board<br>8383 Wilshire Blvd., Suite 302<br>Office of Research Compliance<br>Los Angeles CA, 90211<br>USA
P05216-46	WIRB<br>3535 7th Ave. SW<br>Olympia WA, 98502-5010<br>USA
P05216-47	Western Institutional Review Board<br>3535 7th Ave. SW<br>Olympia WA, 98502-5010<br>USA
P05216-48	Office of Research Integritya<br>19 Hagood Ave - Room 601<br>Charleston SC, 29425<br>USA
P05216-49	UC Davis<br>Office of Research IRB Administration<br>CRISP Building, Suite 1400, Rm 1429<br>2921 Stockton Blvd<br>Sacramento, CA 95817<br>USA
P05216-50	WIRB<br>3535 7th Ave. SW<br>Olympia WA, 98502-5010<br>USA
P05216-51	WIRB<br>3535 7th Ave. SW<br>Olympia WA, 98502-5010<br>USA

P05216-52	University of Miami<br>Human Subjects Research Office<br>1500 NW 12th Ave Suite 1000<br>Human Subjects Research Office<br>Suite 1000<br>Miami FL, 33136<br>USA
P05216-53	University of Cincinnati Medical Center<br>Investigational Review Board<br>51 Goodman Drive Suite 300<br>Cincinnati OH, 45221<br>USA<br><br>University of Cincinnati Medical Center<br>Institutional Review Board<br>G-08, Wherry Hall, Eden Ave.<br>P.O. Box 670567<br>Cincinnati, OH 45267-0567
P05216-54	McGuire Institutional Review Board<br>McGuire DVAMC<br>1201 Broad Rock Blvd. Room 3C-126<br>Richmond VA, 23249<br>USA<br><br>Western Institutional Review Board<br>3535 Seventh Avenue SW<br>Olympia WA, 98502<br>USA
P05216-55	Western Institutional Review Board<br>3535 Seventh Avenue, SW<br>Olympia WA, 98508-2029 98502-5010<br>USA
P05216-56	WIRB<br>3535 7th Ave. Southwest<br>Olympia WA, 98502<br>USA
P05216-57	John Hopkins Medicine Institutional Review Board<br>1620 McElderry Street Reed Hall B-130<br>Reed Hall B-130<br>Baltimore MD, 21205-1911<br>USA
P05216-58	Baylor College of Medicine<br>One Baylor Plaza Rm 600 D<br>Office of Research<br>Rm 600 D<br>Houston TX, 77030-3411<br>USA
P05216-59	WIRB<br>3535 7th Ave. Southwest<br>Olympia WA, 98502-5010<br>USA
P05216-63	Ethik-Kommission des Fachbereichs Medizin der<br>Johann Wolfgang Goethe-Universitaet<br>Theodor-Stern-Kai 7

Protocol and Center Number	Responsible IRB Name and Address(es)
	Frankfurt am Main<br>Frankfurt 60590<br>Germany
P05216-64	Ethik-Kommission des Fachbereichs Medizin der<br>Johann Wolfgang Goethe-Universitaet<br>Frankfurt am Main<br>Theodor-Stern-Kai 7<br>Frankfurt 60590<br>Germany<br><br>Landesamt fuer Gesundheit und Soziales Berlin<br>Geschaeftsstelle der Ethik-Kommission des<br>Saechsische Strasse 28<br>Landes Berlin<br>Berlin 10707<br>Germany
P05216-65	Ethik-Kkommission der Aerztekammer Nordrhein<br>Tersteegenstrasse 31<br>Duesseldorf 40474<br>Germany<br><br>Ethik-Kommission des Fachbereichs Medizin der<br>Johann Wolfgang Goethe-Universitaet<br>Theodor-Stern-Kai 7<br>Frankfurt am Main<br>Frankfurt 60590<br>Germany
P05216-66	Ethik-Kommission der Landesaerztekammer<br>Rheinland-Pfalz<br>Deutschhausplatz 3<br>Mainz 55116<br>Germany<br><br>Ethik-Kommission des Fachbereichs Medizin der<br>Johann Wolfgang Goethe-Universitaet<br>Theodor-Stern-Kai 7<br>Frankfurt am Main<br>Frankfurt 60590<br>Germany
P05216-67	Ethik-Kommission der Medizinischen Fakultaet der<br>Technischen Universitaet Muenchen<br>Ismaninger Str. 22<br>Muenchen 81675<br>Germany<br><br>Ethik-Kommission des Fachbereichs Medizin der<br>Johann Wolfgang Goethe-Universitaet<br>Theodor-Stern-Kai 7<br>Frankfurt am Main<br>Frankfurt 60590<br>Germany
P05216-68	Ethik-Kommission der Medizinischen Fakultaet der<br>Universitaet zu Koeln<br>Joseph-Stelzmann-Str. 20<br>Koeln 50931<br>Germany<br><br>Ethik-Kommission des Fachbereichs Medizin der<br>Johann Wolfgang Goethe-Universitaet

Protocol and Center Number	Responsible IRB Name and Address(es)
	Theodor-Stern-Kai 7<br>Frankfurt am Main<br>Frankfurt 60590<br>Germany
P05216-69	Ethik-Kkommission der Medizinischen Fakultaet Heidelberg<br>Alte Glockengiesserei 11/1<br>Heidelberg 69115<br>Germany<br><br>Ethik-Kommission des Fachbereichs Medizin der Johann Wolfgang Goethe-Universitaet<br>Frankfurt am Main<br>Theodor-Stern-Kai 7<br>Frankfurt 60590<br>Germany
P05216-70	Ethik-Kommission der Med. Fakultaet der Universitaet Duisburg-Essen<br>Universitaetsklinikum Essen<br>Robert-Koch-Str. 9-11<br>Essen 45147<br>Germany<br><br>Ethik-Kommission des Fachbereichs Medizin der Johann Wolfgang Goethe-Universitaet<br>Theodor-Stern-Kai 7<br>Frankfurt am Main<br>Frankfurt 60590<br>Germany
P05216-71	Azienda Ospedaliera Universitaria Policlinico Paolo Giaccone<br>Via del Vespro, 127<br>Comitato Etico<br>Palermo 90127<br>Italy
P05216-72	Comitato Etico- A.O. San Giovanni Battista - Le Molinette<br>Corso Bramante, 88<br>Torino 10126<br>Italy
P05216-73	Azienda Ospedaliera Spedali Civili di Brescia<br>Comitato Etico<br>Piazzale Spedali Civili, 1<br>Comitato Etico<br>Brescia 25123<br>Italy
P05216-74	Azienda Ospedaliero-Universitaria Careggi<br>Comitato Etico<br>Viale Pieraccini, 28<br>Comitato Etico<br>Firenze 50139<br>Italy<br><br>Azienda Ospedaliero-Universitaria Careggi<br>CTO, Largo Palagi 1<br>Firenze 50139<br>Italy

Protocol and Center Number	Responsible IRB Name and Address(es)
P05216-75	Azienda Ospedaliero (A.O.U.) Maggiore della Carita di Novara<br>Comitato Etico Interaziendale<br>Corso Mazzini 18<br>Novara, 28100<br>Italy
P05216-76	Policlinico Umberto<br>Comitato Etico<br>Viale del Policlinico, 155<br>Comitato Etico<br>Roma 00161<br>Italy
P05216-77	Azienda Ospedaliera Universitaria Policlinico Tor Vergata<br>Viale Oxford, 81<br>Comitato Etico<br>Roma 00133<br>Italy
P05216-78	Ospedale Casa Sollievo della Sofferenza-Istituto di Ricovero e Cura a Carattere Scientifico<br>Comitato Etico-Casa Sollievo Della Sofferenza<br>Viale Cappuccini 1<br>San Giovanni Rotondo (Foggia), 71013<br>Italy
P05216-79	Hospital Vall D`Hebron<br>Passeig Vall d'Hebron 119-129 08035<br>CEIC<br>Institut de Recerca, Planta 2<br>Barcelona<br>Spain
P05216-80	Hospital 12 de Octubre<br>Comite Etico de Investigacion Clinica<br>Ctra. de Andalucia - Km 5,4<br>Madrid 28041<br>Spain<br><br>Hospital Vall D`Hebron<br>Passeig Vall d'Hebron 119-129 08035<br>CEIC<br>Institut de Recerca, Planta 2<br>Barcelona<br>Spain
P05216-81	Hospital Puerta de Hierro<br>San Martin de Porres, 4<br>Comite Etico de Investigacion Clinica<br>Madrid 28035<br>Spain<br><br>Hospital Puerta de Hierro de Majadahonda<br>Comite Etico de Investigacion Clinica,<br>planta 1<br>C/ Manuel de Falla, 1 planta 1<br>Majadahonda, Madrid 28222<br>Spain<br><br>Hospital Vall D`Hebron<br>Passeig Vall d'Hebron 119-129 08035<br>CEIC

Protocol and Center Number	Responsible IRB Name and Address(es)
	Institut de Recerca, Planta 2<br>Barcelona<br>Spain
P05216-82	Hospital General Universitario de Valencia<br>Comite Etico de Investigacion Clinica<br>Avda. Tres Cruces, s/n<br>Valencia 46014<br>Spain<br><br>Hospital Vall d'Hebron<br>Comite Etico de Investigacion Clinica<br>Institut de Recerca<br>Passeig de Vall d'Hebron 119 – 129<br>Barcelona, 08035<br>Spain
P05216-83	Hospital Clinic i Provincial<br>Comite Etico de Investigacion Clinica<br>Villarroel, 170<br>Barcelona, 08036<br>Spain<br><br>Hospital Vall D`Hebron<br>Passeig Vall d'Hebron 119-129 08035<br>CEIC<br>Institut de Recerca, Planta 2<br>Barcelona<br>Spain
P05216-84	Hospital Clinico Universitario de Valencia<br>Comite Etico de Investigacion Clinica<br>Avda. Blasco Ibanez, 17<br>Comite Etico de Investigacion Clinica<br>Valencia 46010<br>Spain<br><br>Hospital Vall D`Hebron<br>Passeig Vall d'Hebron 119-129 08035<br>CEIC<br>Institut de Recerca, Planta 2<br>Barcelona<br>Spain
P05216-85	Comite Etico de Investigacion Clinica H. La Princesa<br>Diego de Leon, 62<br>Madrid 28006<br>Spain<br><br>Hospital Vall D`Hebron<br>Passeig Vall d'Hebron 119-129 08035<br>CEIC<br>Institut de Recerca, Planta 2<br>Barcelona<br>Spain
P05216-86	Fundacion Hospital Alcorcon<br>Comite Etico de Investigacion Clinica<br>Alcorcon<br>Budapest, 1<br>Madrid 28922<br>Spain

Protocol and Center Number	Responsible IRB Name and Address(es)
	Hospital Vall d'Hebron<br>Comite Etico de Investigacion Clinica<br>Passeig de Vall d'Hebron 119 – 129<br>Barcelona, 08035<br>Spain
P05216-90	Hopital Erasme ULBComite d'Ethique<br>Route de Lennik 808<br>Bruxelles 1070<br>Belgium<br><br>UZ Gasthuisberg<br>Commissie voor Medische Ethiek / Klinisch Onderzoek<br>Herestraat, 49Leuven 3000<br>Belgium
P05216-91	UZ GasthuisbergCommissie voor Medische Ethiek / Klinisch Onderzoek<br>Herestraat, 49<br>Leuven 3000<br>Belgium
P05216-92	University Health Network<br>Research Ethics Board<br>700 University Avenue<br>Toronto ON, M5G 1Z5<br>Canada
P05216-93	Institutional Review Board Services<br>372 Hollandview Trail, suite 300<br>Aurora ON, L4G 0A5<br>Canada
P05216-94	Mount Sinai Hospital REB<br>600 University Avenue, Rm 1003B<br>Toronto ON, M5G 1X5<br>Canada
P05216-95	Institutional Review Board Services<br>372 Hollandview Trail, suite 300<br>Aurora ON, L4G 0A5<br>Canada
P05216-96	UWO<br>Office of Research Ethics<br>1393 Western Road, Room 4180<br>London ON, N6A 5C1<br>Canada<br><br>UWO<br>London Health Sciences Centre<br>University Campus Site<br>339 Windemere<br>London ON, N6A 5A5<br>Canada
P05216-97	Health Research Ethics Board<br>Biomedical Panel<br>213 Heritage Medical Research Building<br>Edmonton AB, T6G 2S2<br>Canada

Protocol and Center Number	Responsible IRB Name and Address(es)
	Health Research Ethics Board<br>308 Campus Tower<br>Edmonton AB, T6G 0K8<br>Canada
P05216-99	University of Puerto Rico - Medical Science Campus<br>PO Box 365067<br>Institutional Review Board<br>San Juan 00936-5067<br>Puerto Rico<br><br>Western Institutional Review Board<br>3535 7th Ave. SW<br>Olympia WA, 98502-5010<br>USA
P05216-100	Western Institutional Review Board<br>3535 7th Ave. SW<br>Olympia WA, 98502-5010<br>USA
P05216-101	Western Institutional Review Board<br>3535 7th Ave. SW<br>Olympia WA, 98502-5010<br>USA
P05216-102	Academic Medical Center, Medical Ethical Committee<br>Meibergdreef 9<br>Amsterdam 1105 AZ<br>Netherlands
P05216-106	Ethik-Kommission der Med. Fakultaet der Universitaet Tuebingen<br>Schleichstrasse 8<br>Tuebingen 72076<br>Germany<br><br>Ethik-Kommission des Fachbereichs Medizin der Johann Wolfgang Goethe-Universitaet Frankfurt am Main<br>Theodor-Stern-Kai 7<br>Frankfurt 60590<br>Germany
P05216-107	Ethikkommission der Universitaet Ulm<br>Helmholtzstr. 20 (Oberer Eselsberg)<br>Ulm 89081<br>Germany<br><br>Ethik-Kommission des Fachbereichs Medizin der Johann Wolfgang Goethe-Universitaet Frankfurt am Main<br>Theodor-Stern-Kai 7<br>Frankfurt 60590<br>Germany
P05216-108	Ethik-Kommission. Universitaet. zu Luebeck<br>Ratzeburger Allee 160<br>Luebeck 23538<br>Germany<br><br>Ethik-Kommission des Fachbereichs Medizin der Johann Wolfgang Goethe-Universitaet Frankfurt am Main

Protocol and Center Number	Responsible IRB Name and Address(es)
	Theodor-Stern-Kai 7<br>Frankfurt 60590<br>Germany
P05216-109	Ethik-Kommission der Albert-Ludwigs-Universitaet Freiburg<br>Elsaesser Strasse 2m, Haus 1a<br>Freiburg 79110<br>Germany<br><br>Ethik-Kommission des Fachbereichs Medizin der Johann Wolfgang Goethe-Universitaet<br>Frankfurt am Main<br>Theodor-Stern-Kai 7<br>Frankfurt 60590<br>Germany
P05216-110	Ethikkommission der Medizinischen Fakulaet der Universitaet Wuerzburg<br>Josef-Schneider-Str. 2, D7<br>Wuerzburg 97080<br>Germany<br><br>Ethik-Kommission des Fachbereichs Medizin der Johann Wolfgang Goethe-Universitaet<br>Frankfurt am Main<br>Theodor-Stern-Kai 7<br>Frankfurt 60590<br>Germany
P05216-111	The Ottawa Hospital Research Ethics Board<br>Ottawa Hospital Civic Campus<br>751 Parkdale Avenue, Suite 106<br>Ottawa ON, K1Y 1J7<br>Canada
P05216-112	Clinical Research Ethics Board<br>828 West 10th Ave Rm 210<br>Vancouver BC, V6K 1L8<br>Canada<br><br>The Office of Research Services<br>102-6190 Agronomy Road Vancouver BC, V6T 1Z3<br>Canada
P05216-113	Health Research Ethics Board<br>308 Campus Tower<br>Edmonton AB,<br>Canada<br><br>Health Research Ethics Board<br>Biomedical Panel<br>213 Heritage Medical Research Building<br>Edmonton AB, T6G 2S2<br>Canada
P05216-114	CHUM - Hopital Notre-Dame<br>3981 St. Laurent blvd., Mezz 2, Rm M-209<br>Montreal QC, H2W 1Y5<br>Canada
P05216-115	University Health Network REB<br>Research Ethics Board

Protocol and Center Number	Responsible IRB Name and Address(es)
	700 University Avenue<br>Toronto ON, M5G 1Z5<br>Canada
P05216-116	MUHC - Royal Victoria Hospital<br>Research Ethics Board<br>687 Pine Avenue West, Room #511-08<br>Montreal QC, H3A 1A1<br>Canada
P05216-117	Ospedale Fatebenefratelli e Oftalmico di Milano<br>Comitato Etico Indipendente<br>Corso di Porta Nuova 23<br>Milano<br>Italy
P05216-119	CPP "Sud-Est II"<br>Hopital Hotel Dieu<br>1 Place de l'Hopital, Porte 16<br>Lyon Cedex 02 69288<br>France
P05216-120	CPP "Sud-Est II"<br>Hopital Hotel Dieu<br>1 Place de l'Hopital, Porte 16<br>Lyon Cedex 02 69288<br>France
P05216-121	CPP "Sud-Est II"<br>1 Place de l'Hopital, Porte 16 Hopital Hotel Dieu<br>Hopital Hotel Dieu<br>Lyon Cedex 02 69288<br>France
P05216-122	CPP "Sud-Est II"<br>Hopital Hotel Dieu<br>1 Place de l'Hopital, Porte 16<br>Lyon Cedex 02 69288<br>France
P05216-123	CPP "Sud-Est II"<br>Hopital Hotel Dieu<br>1 Place de l'Hopital, Porte 16<br>Lyon Cedex 02 69288<br>France
P05216-124	IRB for Health Sciences Research<br>University of Virginia<br>One Morton Drive Suite 400<br>Charlottesville VA, 22908<br>USA
P05216-125	Biomedical Research Alliance of New York<br>Institutional Review Board<br>1981 Marcus Avenue, Suite 210<br>Lake Success NY, 11042<br>USA<br><br>Biomedical Research Alliance of New York, LLC<br>Institutional Review Board<br>225 Communirty Drive., Suite 100<br>Great Neck NY, 11021<br>USA

Protocol and Center Number	Responsible IRB Name and Address(es)
P05216-127	Washington University School of Medicine<br>Human Research Protection Office<br>22 North Euclid Avenue Suite 233<br>Suite 233<br>St. Louis MO, 63108<br>USA
P05216-128	Western Institutional Review Board<br>3535 Seventh Avenue, SW<br>Olympia WA, 98502SA
P05216-129	Institutional Review Board<br>Baylor Research Institute<br>3310 Live Oak Street Suite 501<br>Dallas TX, 75204<br>USA
P05216-130	University of Maryland<br>Institutional Review Board<br>655 West Baltimore Street<br>Baltimore MD, 21201<br>USA
P05216-131	Lahey Clinic IRB<br>Lahey Clinic<br>41 Mall Road<br>Burlington MA, 01805<br>USA
P05216-132	Mayo Clinic IRB<br>201 Building 440<br>200 First Street SW<br>Rochester NY, 55905<br>USA
P05216-133	Brooke Army Medical Center IRB<br>Dept of Clinical Investigations<br>3400 Rawley E. Chambers, Suite A<br>San Antonio TX, 78234<br>USA
P05216-134	Maine Medical Center Institutional Review Board<br>81 Research Drive<br>Scarborough ME, 04074<br>USA
P05216-135	University of Kentucky IRB<br>315 Kinkead Hall<br>Lexington KY, 40506-0057<br>USA
P05216-136	University of Vermont Institutional Review Board<br>231 Rowell Building<br>Burlington VT, 05405<br>USA<br><br>University of Vermont Institutional Review Board<br>245 South Park<br>Colchester VT, 05466<br>USA
P05216-138	SUNY Upstate Medical University Institutional Review Board<br>750 East Adams Street 1254 Weiskotten Hall<br>for the Protection of Human Subjects<br>1254 Weiskotten Hall

Protocol and Center Number	Responsible IRB Name and Address(es)
	Syracuse NY, 13210<br>USA
P05216-140	University of Michigan Medical School IRB<br>517 W. William Argus 1<br>Argus 1<br>Ann Arbor MI, 48103<br>USA<br><br>University of Michigan Medical School<br>Insitutional Reivew Board for Human Subject Research<br>2800 Plymouth, Building 200, Room 2086<br>Ann Arbor, MI 48109 – 2800<br>USA
P05216-142	The Nebraska Medical Center Institutional Review Board<br>987830 The Nebraska Medical Center ARS 3000<br>Office of Regulatory Affairs<br>ARS 3000<br>Omaha NE, 68198<br>USA
P05216-143	WIRB<br>3535 7th Ave. Southwest<br>Olympia WA, 98502<br>USA
P05216-144	WIRB<br>3535 7th Ave. Southwest<br>Olympia WA, 98502<br>USA
P05216-145	Western Institutional Review Board<br>3535 Seventh Ave., SW<br>Olympia WA, 98502<br>USA
P05216-146	Western Institutional Review Board<br>3535 Seventh Avenue, SW<br>Olympia WA, 98502<br>USA
P05216-147	Kaiser Permanente Northern IRB (KPNC IRB)<br>1800 Harris 16th Floor<br>Oakland CA, 94612<br>USA
P05216-150	Kaiser Permanente of the Mid-Atlantic States<br>2101 East Jefferson Street<br>Institutional Review Board<br>Rockville MD, 20852<br>USA
P05216-152	Western Institutional Review Board<br>3535 Seventh Avenue, SW<br>Olympia WA, 98502<br>USA
P05216-153	Western Institutional Review Board<br>3535 7th Avenue Southwest PO Box 12029<br>Olympia WA, 98502-5010<br>USA
P05216-156	Hennepin County Medical Center

Protocol and Center Number	Responsible IRB Name and Address(es)
	Minn. Med Rsrch FNDN Human Subjects Research Committee<br>914 So 8th St<br>900 HFA Bldg<br>Minneapolis MN, 55404<br>USA
P05216-157	University of Pennsylvania<br>3624 Market Street Suite 301S<br>Institutional Review Board<br>Philadelphia PA, 19104<br>USA
P05216-159	CHU Brugmann<br>Place Arthur Van Gehuchten, 4 Ethics Committee<br>Site Victor Horta<br>Ethics Committee<br>Bruxelles 1020<br>Belgium<br><br>UZ Gasthuisberg<br>Herestraat, 49<br>Commissie voor Medische Ethiek / Klinisch Onderzoek<br>Leuven 3000<br>Belgium
P05216-161	Hospital Ramon y Cajal<br>Ctra. de Colmenar Viejo, Km. 9,1<br>Comite Etico de Investigacion Clinica<br>Madrid, 28034<br>Spain<br><br>Hospital Vall d'Hebron<br>Passeig de Vall d'Hebron 119 – 129<br>Comite Etico de Investigacion Clinica<br>Barcelona, 08035<br>Spain
P05216-162	Hospital Universitario Reina Sofia<br>Avda. Menendez Pidal, s/n<br>Comite Etico de Investigacion Clinica<br>Cordoba 14004<br>Spain<br><br>Hospital Vall D`Hebron<br>Passeig Vall d'Hebron 119-129 08035 Institut de Recerca, Planta 2<br>CEIC<br>Institut de Recerca, Planta 2<br>Barcelona<br>Spain
P05216-166	Western Institutional Review Board<br>3535 Seventh Avenue, SW<br>Olympia WA, 98502<br>USA
P05216-168	WIRB<br>3535 7th Ave. Southwest<br>Olympia WA, 98502<br>USA
P05216-170	Western Institutional Review Board<br>3535 Seventh Ave, SW

Protocol and Center Number	Responsible IRB Name and Address(es)
	Olympia WA, 98502<br>USA
P05216-171	Western Institutional Review Board<br>3535 Seventh Avenue, SW<br>Olympia WA, 98502<br>USA
P05216-174	WIRB<br>3535 7th Ave. SW<br>Olympia WA, 98502-5010<br>USA
P05216-175	University of California Irvine<br>4199 Campus Dr., Ste 300 Office of Research Admin<br>Human Research Protections Program<br>Irvine CA, 92697-7600<br>USA
P05216-176	Mayo Clinic<br>200 First Street SW<br>Institutional Review Board<br>Rochester MN, 55905<br>USA
P05216-178	Kansas City VA Medical Center<br>4801 E. Linwood Blvd. 151<br>IRB<br>Kansas City MO, 64128<br>USA
P05216-179	Kaiser Permanente Southern California<br>393 East Walnut St., 2nd Floor<br>Institutional Review Board<br>Pasadena CA, 91188<br>USA
P05216-180	Walter Reed Army Medical Center<br>6900 Georgia Ave., NW<br>Human Use Committee<br>Washington DC, 20307<br>USA
P05216-181	Western Institutional Review Board<br>3535 Seventh Avenue, SW<br>Olympia WA, 98502<br>USA
P05216-182	Western Institutional Review Board<br>3535 7th Ave. SW<br>Olympia WA, 98502<br>USA
P05216-183	St. John's Institutional Review Board<br>1235 East Cherokee Street<br>Springfield MO, 65804<br>USA
P05216-184	Kaiser Permanente Northern IRB (KPNC IRB)<br>1800 Harris 16th Floor<br>Oakland CA, 94612<br>USA
P05216-185	Western Institutional Review Board<br>3535 Seventh Avenue, SW<br>Olympia WA, 98502<br>USA

Protocol and Center Number	Responsible IRB Name and Address(es)
P05216-186	Comite de Docencia e investigacion - Fundacion CIDEA<br>Billinghurst 1677<br>Buenos Aires C1425DTG<br>Argentina<br><br>Comite Independiente de Etica para Ensayos en Farmacologia Clinica<br>J. E. Uriburu 774 Piso 1<br>Fundacion de Estudios Farmacologicos y de Medicamentos<br>Buenos Aires (C1027AAP)<br>Argentina
P05216-187	Comite de Docencia e Investigacion del Hospital Provincial del Centenario<br>Urquiza 3101<br>Rosario. Santa Fe S2002KDS<br>Argentina
P05216-188	Comite de Bioetica Universitario Austral<br>Av. Juan Domingo Peron 1500 Facultad de Cs Biomedicas<br>Unidad de Investigacion<br>Facultad de Cs Biomedicas<br>Pilar<br>Prov. de Buenos Aires B1629ODT<br>Argentina
P05216-189	Comite de Revision Institucional Hospital Britanico<br>Perdriel 74<br>Ciudad de Buenos Aires C1280AEB<br>Argentina
P05216-190	Comite Independiente de Etica para Ensayos en Farmacologia Clinica<br>J. E. Uriburu 774 Piso 1<br>Fundacion de Estudios Farmacologicos y de Medicamentos<br>Bs As C1027AAP<br>Argentina
P05216-193	CPP "Sud-Est II"<br>1 Place de l'Hopital, Porte 16<br>Hopital Hotel Dieu<br>Lyon Cedex 02 69288<br>France
