# Supplementary material for: Boceprevir for previously untreated patients with chronic hepatitis C Genotype 1 infection: a US-based cost-effectiveness modeling study
Source: BMC Infect Dis. 2013 Apr 27;13:190. doi: 10.1186/1471-2334-13-190 (PMC3643851; doi:10.1186/1471-2334-13-190)
Supplement: Additional file 2: Table S2 — Summary of Costs, QALYs, and ICERs of One-Way Sensitivity Analyses. BOC/RGT vs PR48 and BOC/PR48 vs. PR48. [file 1471-2334-13-190-S2.pdf]

		PR48		BOC/RGT			BOC/PR48			
		Costs	QALYs	Costs	QALYs	ICER: BOC/RGT vs. PR48	Costs	QALYs	ICER: BOC/PR48 vs. PR48	ICER: BOC/PR48 vs. BOC/RGT
Base		58,761	14.55	69,110	15.17	16,792	94,488	15.2	55,162	807,804
**Transition Rates Between Health States**										
F0 to F1	lower bound	58,746	14.55	69,101	15.17	16,811	94,480	15.2	55,202	808,681
	upper bound	58,775	14.55	69,118	15.17	16,776	94,495	15.2	55,126	807,019
F1 to F2	lower bound	58,265	14.58	68,815	15.18	17,448	94,216	15.21	56,633	842,247
	upper bound	59,242	14.52	69,395	15.15	16,174	94,752	15.18	53,770	776,361
F2 to F3	lower bound	58,322	14.58	68,849	15.18	17,407	94,248	15.21	56,580	841,975
	upper bound	58,488	14.57	68,948	15.18	17,173	94,338	15.21	56,040	828,816
F3 to F4	lower bound	58,360	14.59	68,872	15.19	17,485	94,269	15.22	56,912	853,238
	upper bound	59,140	14.51	69,334	15.15	16,161	94,695	15.18	53,564	768,316
F4 to DC	lower bound	56,228	14.71	67,608	15.26	20,595	93,102	15.28	63,912	1,045,399
	upper bound	59,851	14.48	69,755	15.12	15,364	95,084	15.16	51,876	733,057
F4 to HCC	lower bound	57,212	14.76	68,193	15.29	20,689	93,643	15.31	65,908	1,158,357
	upper bound	61,374	14.15	70,652	14.93	11,923	95,909	14.98	41,730	511,581
DC to HCC	lower bound	59,003	14.57	69,266	15.18	16,802	94,635	15.21	55,535	780,430
	upper bound	58,681	14.55	69,058	15.16	16,789	94,440	15.19	55,041	919,793
SVR to DC	lower bound	58,576	14.56	68,798	15.19	16,342	94,162	15.22	54,080	793,663
	upper bound	59,366	14.5	70,123	15.09	18,422	95,549	15.11	59,165	852,064
SVR to HCC	lower bound	58,703	14.56	69,012	15.18	16,600	94,386	15.21	54,647	823,240
	upper bound	58,926	14.53	69,387	15.13	17,364	94,778	15.16	56,709	802,859
DC to LT	lower bound	58,294	14.54	68,811	15.16	17,011	94,208	15.19	55,263	802,888
	upper bound	59,942	14.56	69,864	15.18	16,234	95,196	15.21	54,907	820,721
HCC to LT	lower bound	57,627	14.53	68,401	15.15	17,255	93,826	15.19	55,124	832,153
	upper bound	60,988	14.59	70,501	15.19	15,848	95,787	15.22	55,245	806,193
DC, 1st year to LD	lower bound	59,581	14.57	69,631	15.18	16,541	94,976	15.21	55,474	835,845
	upper bound	58,705	14.55	69,074	15.17	16,809	94,455	15.2	55,141	778,906
DC, subsequent years to LD	lower bound	59,728	14.58	69,729	15.18	16,497	95,069	15.21	55,519	786,973
	upper bound	57,699	14.52	68,430	15.15	17,107	93,851	15.18	54,779	852,595
HCC to LD	lower bound	60,204	14.58	70,008	15.18	16,179	95,326	15.21	55,202	836,189
	upper bound	55,823	14.49	67,283	15.13	17,980	92,785	15.17	55,075	755,483
LT, 1st year to LD	lower bound	58,881	14.55	69,185	15.17	16,752	94,559	15.2	55,195	810,896
	upper bound	58,113	14.53	68,699	15.16	17,007	94,104	15.19	54,984	791,441

LT, subsequent years to LD	lower bound	59,038	14.56	69,287	15.17	16,700	94,655	15.2	55,234	814,670
	upper bound	58,187	14.54	68,743	15.16	16,981	94,144	15.19	55,011	793,718
**Cost Inputs**										
F0	lower bound	58,727	14.55	69,086	15.17	16,809	94,465	15.2	55,180	807,846
	upper bound	58,796	14.55	69,134	15.17	16,775	94,511	15.2	55,144	807,761
F1	lower bound	58,081	14.55	68,650	15.17	17,152	94,056	15.2	55,546	808,678
	upper bound	59,442	14.55	69,569	15.17	16,433	94,920	15.2	54,777	806,930
F2	lower bound	58,307	14.55	68,827	15.17	17,071	94,225	15.2	55,458	808,443
	upper bound	59,216	14.55	69,393	15.17	16,514	94,751	15.2	54,866	807,164
F3	lower bound	58,047	14.55	68,680	15.17	17,253	94,090	15.2	55,651	808,833
	upper bound	59,476	14.55	69,540	15.17	16,331	94,886	15.2	54,673	806,774
F4	lower bound	57,824	14.55	68,543	15.17	17,394	93,963	15.2	55,798	809,125
	upper bound	59,698	14.55	69,676	15.17	16,191	95,013	15.2	54,525	806,482
DC, 1st year	lower bound	58,464	14.55	68,922	15.17	16,970	94,313	15.2	55,350	808,193
	upper bound	59,058	14.55	69,297	15.17	16,615	94,663	15.2	54,974	807,414
DC, subsequent years	lower bound	58,003	14.55	68,628	15.17	17,242	94,037	15.2	55,637	808,782
	upper bound	59,520	14.55	69,591	15.17	16,343	94,939	15.2	54,687	806,826
HCC, 1st year	lower bound	58,110	14.55	68,706	15.17	17,195	94,112	15.2	55,588	808,685
	upper bound	59,413	14.55	69,513	15.17	16,390	94,864	15.2	54,736	806,922
HCC, subsequent years	lower bound	58,152	14.55	68,731	15.17	17,167	94,135	15.2	55,558	808,620
	upper bound	59,371	14.55	69,488	15.17	16,418	94,841	15.2	54,766	806,987
LT, 1st year	lower bound	58,495	14.55	68,943	15.17	16,954	94,332	15.2	55,333	808,156
	upper bound	59,028	14.55	69,277	15.17	16,631	94,644	15.2	54,991	807,451
LT, subsequent years	lower bound	58,306	14.55	68,821	15.17	17,063	94,218	15.2	55,448	808,387
	upper bound	59,217	14.55	69,398	15.17	16,522	94,758	15.2	54,876	807,220
SVR	lower bound	58,761	14.55	69,110	15.17	16,792	94,488	15.2	55,162	807,804
	upper bound	62,276	14.55	75,003	15.17	20,651	100,659	15.20	59,262	816,639
**Utility Values**										
F0	lower bound	58,761	14.53	69,110	15.16	16,667	94,488	15.19	54,707	788,844
	upper bound	58,761	14.56	69,110	15.17	16,891	94,488	15.21	55,521	823,193
F1	lower bound	58,761	14.25	69,110	14.96	14,491	94,488	15.01	46,995	550,665
	upper bound	58,761	14.78	69,110	15.33	19,159	94,488	15.35	63,782	1,268,520
F2	lower bound	58,761	14.35	69,110	15.04	14,977	94,488	15.08	48,825	621,989
	upper bound	58,761	14.7	69,110	15.26	18,540	94,488	15.29	61,356	1,052,315
F3	lower bound	58,761	14.4	69,110	15.08	15,297	94,488	15.11	49,972	660,335

	upper bound	58,761	14.67	69,110	15.24	18,174	94,488	15.26	60,010	977,611
F4	lower bound	58,761	14.38	69,110	15.07	15,163	94,488	15.1	49,520	650,573
	upper bound	58,761	14.74	69,110	15.28	19,068	94,488	15.3	63,157	1,104,363
SVR, F0	lower bound	58,761	14.53	69,110	15.13	17,175	94,488	15.16	56,500	851,247
	upper bound	58,761	14.55	69,110	15.17	16,792	94,488	15.2	55,162	807,804
SVR, F1	lower bound	58,761	14.23	69,110	14.64	25,685	94,488	14.64	87,264	3,896,337
	upper bound	58,761	14.55	69,110	15.17	16,792	94,488	15.2	55,162	807,804
SVR, F2	lower bound	58,761	14.47	69,110	15.03	18,388	94,488	15.06	60,766	1,008,131
	upper bound	58,761	14.55	69,110	15.17	16,792	94,488	15.2	55,162	807,804
SVR, F3	lower bound	58,761	14.53	69,110	15.13	17,175	94,488	15.16	56,500	851,247
	upper bound	58,761	14.55	69,110	15.17	16,792	94,488	15.2	55,162	807,804
SVR, F4	lower bound	58,761	14.53	69,110	15.13	17,158	94,488	15.16	56,440	849,258
	upper bound	58,761	14.55	69,110	15.17	16,792	94,488	15.2	55,162	807,804
DC, 1st year	lower bound	58,761	14.54	69,110	15.16	16,671	94,488	15.19	54,741	795,149
	upper bound	58,761	14.56	69,110	15.17	16,899	94,488	15.2	55,533	819,139
DC, subsequent years	lower bound	58,761	14.52	69,110	15.15	16,492	94,488	15.18	54,119	777,011
	upper bound	58,761	14.58	69,110	15.18	17,063	94,488	15.21	56,102	836,635
HCC, 1st year	lower bound	58,761	14.53	69,110	15.16	16,630	94,488	15.19	54,600	790,974
	upper bound	58,761	14.56	69,110	15.17	16,931	94,488	15.21	55,643	822,504
HCC, subsequent years	lower bound	58,761	14.54	69,110	15.16	16,642	94,488	15.19	54,641	792,246
	upper bound	58,761	14.56	69,110	15.17	16,920	94,488	15.21	55,607	821,352
LT, 1st year	lower bound	58,761	14.55	69,110	15.17	16,786	94,488	15.2	55,140	807,142
	upper bound	58,761	14.55	69,110	15.17	16,800	94,488	15.2	55,190	808,656
LT, subsequent years	lower bound	58,761	14.55	69,110	15.16	16,753	94,488	15.2	55,025	803,722
	upper bound	58,761	14.56	69,110	15.17	16,843	94,488	15.2	55,339	813,112
AV Therapy, no side effects	lower bound	58,761	14.52	69,110	15.14	16,669	94,488	15.16	55,499	1,107,530
	upper bound	58,761	14.58	69,110	15.19	16,918	94,488	15.23	54,829	635,753
AV Therapy, anemia	lower bound	58,761	14.54	69,110	15.16	16,848	94,488	15.18	55,775	963,132
	upper bound	58,761	14.56	69,110	15.18	16,695	94,488	15.22	54,120	629,999
Male, population norm	lower bound	58,761	14.43	69,110	15.04	16,962	94,488	15.07	55,764	830,067
	upper bound	58,761	14.67	69,110	15.29	16,634	94,488	15.32	54,575	780,823
Female, population norm	lower bound	58,761	14.47	69,110	15.08	16,925	94,488	15.11	55,615	819,529
	upper bound	58,761	14.63	69,110	15.26	16,665	94,488	15.29	54,711	792,135
**Treatment Efficacy**										
SVR,	lower bound	59,838	14.48	69,110	15.17	13,440	94,488	15.2	48,040	807,804

PR48	upper bound	56,185	14.73	69,110	15.17	29,369	94,488	15.2	81,237	807,804
SVR, BOC/RGT	lower bound	58,761	14.55	70,727	15.06	23,664	94,488	15.2	55,162	68,299
	upper bound	58,761	14.55	68,783	15.19	15,694	94,488	15.2	55,162	(818,433)
SVR, BOC/PR 48	lower bound	58,761	14.55	69,110	15.17	16,792	95,595	15.12	64,401	(207,921)
	upper bound	58,761	14.55	69,110	15.17	16,792	92,110	15.36	41,157	108,378
**Discount Rates**										
Costs and QALYs	lower bound	78,349	22.46	81,217	23.69	2,338	106,218	23.79	21,016	251,235
	upper bound	51,878	11.51	64,772	11.93	30,630	90,134	11.94	88,789	2,562,542

PR48 – peginterferon-ribavirin regimen for 48 weeks; BOC/RGT – peginterferon-ribavirin and boceprevir for 24 weeks, and those with a detectable hepatitis C virus (HCV) RNA level between weeks 8 and 24 received peginterferon–ribavirin from week 28 to week 48; BOC/PR48 – peginterferon–ribavirin for 48 weeks and boceprevir for 44 weeks; AV therapy – antiviral therapy; SVR – sustained virologic response; F0 – no fibrosis; F1 – portal fibrosis without septa; F2 – portal fibrosis with few septa; F3 – numerous septa without cirrhosis; F4 – cirrhosis; DC – decompensated cirrhosis; HCC – hepatocellular carcinoma; LT – liver transplant; LD – liver-related death; QALY – quality-adjusted life years; ICER – incremental cost-effectiveness ratios
